# Exploration of adverse event profiles for cefotaxime: a disproportionality analysis using the FDA adverse event reporting system

**DOI:** 10.1186/s40360-025-00960-w

**Published:** 2025-07-01

**Authors:** Cheng Jiang, Jiancheng Qian, Yingyan Lu, Junxian Zheng

**Affiliations:** 1 Zhejiang Academy of Traditional Chinese Medicine, Tongde Hospital of Zhejiang Province, Gucui Road NO. 234, Hangzhou, Zhejiang 310012 China; 2 Zhejiang Provincial Key Laboratory of Disease-Syndrome Integrated Cancer Prevention and Treatment, Tongde Hospital of Zhejiang Province, Hangzhou, Zhejiang 310012 China; 3 Zhejiang Provincial Key Laboratory of Traditional Chinese Medicine for Pharmacodynamic Material Basis Research of Chinese Medicine, Tongde Hospital of Zhejiang Province, Hangzhou, Zhejiang 310012 China

**Keywords:** Cefotaxime, FAERS, Dosing frequency, Hepatobiliary disorder, Subgroup analysis

## Abstract

**Background:**

Cefotaxime has been widely used in the clinical treatment of infections. However, there is still a lack of systematic researches for the adverse event profiles of cefotaxime through large-scale post-marketing monitoring.

**Methods:**

This study investigated the adverse event profiles for cefotaxime in the Food and Drug Administration Adverse Event Reporting System database, delving into clinical characteristics, adverse event signals and variations in these signals across subgroups.

**Results:**

Compared with Asia, Europe and America more commonly reported once-daily frequency. New severe hepatobiliary disorders were observed in neonates, children, or underweight elderly patients even when using adjusted doses below 1 g. Significant variations in adverse event signals were identified in relation to continent, dose, onset time, and outcome.

**Conclusions:**

The existence of non-recommended frequency in Europe and America warrants clinical attention when using cefotaxime. The identification of new severe hepatobiliary disorders highlights the critical need for personalized dosing strategies and intensified liver function monitoring for neonates, children, and elderly individuals with lower body weights. Furthermore, the differences in adverse event signals across subgroups underscore the necessity of developing targeted monitoring protocols. Further research is required to validate the association.

**Clinical trial number:**

Not applicable.

**Supplementary Information:**

The online version contains supplementary material available at 10.1186/s40360-025-00960-w.

## Plain language summary

**Background:**

Cefotaxime is a commonly used antibiotic. This study investigated the post-marketing safety of cefuroxime.

**Research design and methods:**

We analyzed the clinical characteristics of adverse event reports related to cefotaxime using Food and Drug Administration Adverse Event Reporting System database. Then, we detected the adverse events of cefotaxime. We further examined the differences of adverse events across different subgroups.

**Results:**

In comparison to Asia, Europe and America more commonly reported once-daily frequency. Severe hepatobiliary disorders were primarily seen in neonates, children, and elderly individuals with low body weights. Moreover, significant variations in adverse events were observed across continent, dose, onset time, and outcome subgroups.

**Conclusion:**

There was non-recommended frequency in Europe and America. Personalized dosing strategies for neonates, children, and elderly individuals with low body weights, as well as targeted monitoring protocols across subgroups, were important.

## Background

Cefotaxime, the first third-generation cephalosporin antibiotic (synthesized in 1976), is approved by the Food and Drug Administration (FDA) for the treatment of infections caused by Gram-positive, Gram-negative, and anaerobic bacteria [1]. Similar to other agents of third-generation cephalosporin antibiotics, cefotaxime exerts bactericidal effects by inhibiting the activity of bacterial cell wall synthesis enzymes, disrupting the process of cell wall synthesis, and causing cell wall defects [2]. Compared to first and second-generation cephalosporin antibiotics, cefotaxime has a broad spectrum of in vitro activity, including beta-lactamase-producing Escherichia coli [3]. Furthermore, cefotaxime forms a metabolite, desacetylcefotaxime, which not only exhibits antibacterial properties but also penetrates well into various important body compartments [4], including central nervous system (CNS) [5, 6]. Till now, cefotaxime has been widely used in the clinical treatment of localized infections as well as systemic infectious diseases affecting the lower respiratory tract, genitourinary tract, central nervous system, intra-abdominal infections, bone and joint infections, skin infections, gynecologic infections, and septicemia [7–9].

However, cefotaxime still poses potential safety concerns in practical applications. According to the labels for cefotaxime, the most frequently reported adverse reactions include local reactions (4.3%), hypersensitivity reactions (2.4%), and gastrointestinal effects (1.4%). Additionally, serious adverse events have been documented, such as life-threatening cardiac arrhythmias [1], severe cutaneous adverse reactions (SCAR) [10] and drug reaction with eosinophilia and systemic symptom (DRESS) [11]. Despite over four decades of widespread clinical use, systematic post-marketing surveillance studies on cefotaxime’s adverse event profiles remain limited.

The FDA Adverse Event Reporting System (FAERS) is a world-class drug vigilance database that encompasses millions of reports of adverse events submitted by physicians, pharmacists, consumers, and others, serving as an effective tool for detecting adverse event signals related to drug exposure [12]. Till now, it has been utilized for mining adverse event signals of antibiotics such as ertapenem, cefepime, imipenem, ofloxacin, cefuroxime, ceftazidime, cefoxitin, streptomycin, fosfomycin, daptomycin, tigecycline, vancomycin, and linezolid [13–18]. This study investigated the adverse event profiles for cefotaxime in the FAERS database, delving into clinical characteristics, adverse event signals and variations in these signals across subgroups. This study enhances the understanding of cefotaxime’s adverse event profiles and offers valuable insights for the clinical management of cefotaxime.

## Methods

### Data source and collection

All raw data covering the period from the first quarter of 2019 to the fourth quarter of 2023 were downloaded in ASCII format from the FAERS database. Adverse event reports of cefotaxime were identified by searching for the “CEFOTAXIME SODIUM” in the “prod_ai” column. Based on the “role_cod” column, the reports with cefotaxime labeled as the “PS” (primary suspected) were extracted, and other drugs labeled as “SS” (secondary suspect), “C” (concomitant), or “I” (interacting) in these cases were considered as concomitant drugs [19–22]. The standardized Medical Dictionary for Regulatory Activities (MedDRA) version 27.0 was used to classified the Preferred Term (PT) to the corresponding primary system organ class (SOC) [19–22]. The additional details regarding data source and collection can be found in our previously published articles [19–22].

### Statistical analysis

Based on valid data, descriptive analysis was utilized to examine the clinical characteristics of the cefotaxime-associated adverse event reports regarding the reporter (including reporter continent, reporter country and reporter type), patient (including sex, age and weight), product (including dose, frequency and indication), and problem (including report season, onset time and outcome). Subsequently, the relationship between reporter continent, reporter country, age, weight, dose, and frequency of the cefotaxime-associated adverse event reports were analyzed.

The adverse event signals of cefotaxime were explored at the SOC and PT levels using four disproportionality algorithms [19, 20], including reporting odds ratio (ROR) [23, 24], proportional reporting ratio (PRR) [25, 26], Bayesian confidence propagation neural network (BCPNN) [25–27], and the multi-item gamma Poisson shrinker (MGPS) [25–27]. Concomitant medications may potentially confound signal detection analysis. To assess the robustness of adverse event signals, sensitivity analyses were conducted by excluding reports involving frequently co-administered drugs, incorporating a rigorous evaluation of concomitant drug factors. To further examine other potential influencing factors, the clinical characteristics of typical adverse event signals were systematically analyzed across the reporter (including reporter continent, reporter country and reporter type), patient (including sex, age and weight), product (including dose, frequency and indication), and problem (including report season, onset time and outcome).

To evaluate potential variations in adverse event reporting, this study performed in-depth subgroup analyses of adverse event signals based on reporter (reporter continent), patient (sex, age and weight), product (dose and frequency), and problem (onset time and outcome) [19–22]. The equations and criteria of the algorithms can be found in Supplementary Tables S1-S4. All data processing and statistical analyses were performed using Python 3 programming language in Jupyter Notebook version 6.4.12. The additional details regarding statistical analysis can be found in our previously published articles [19–22].

## Results

### Clinical characteristics analysis

**Fig. 1:**
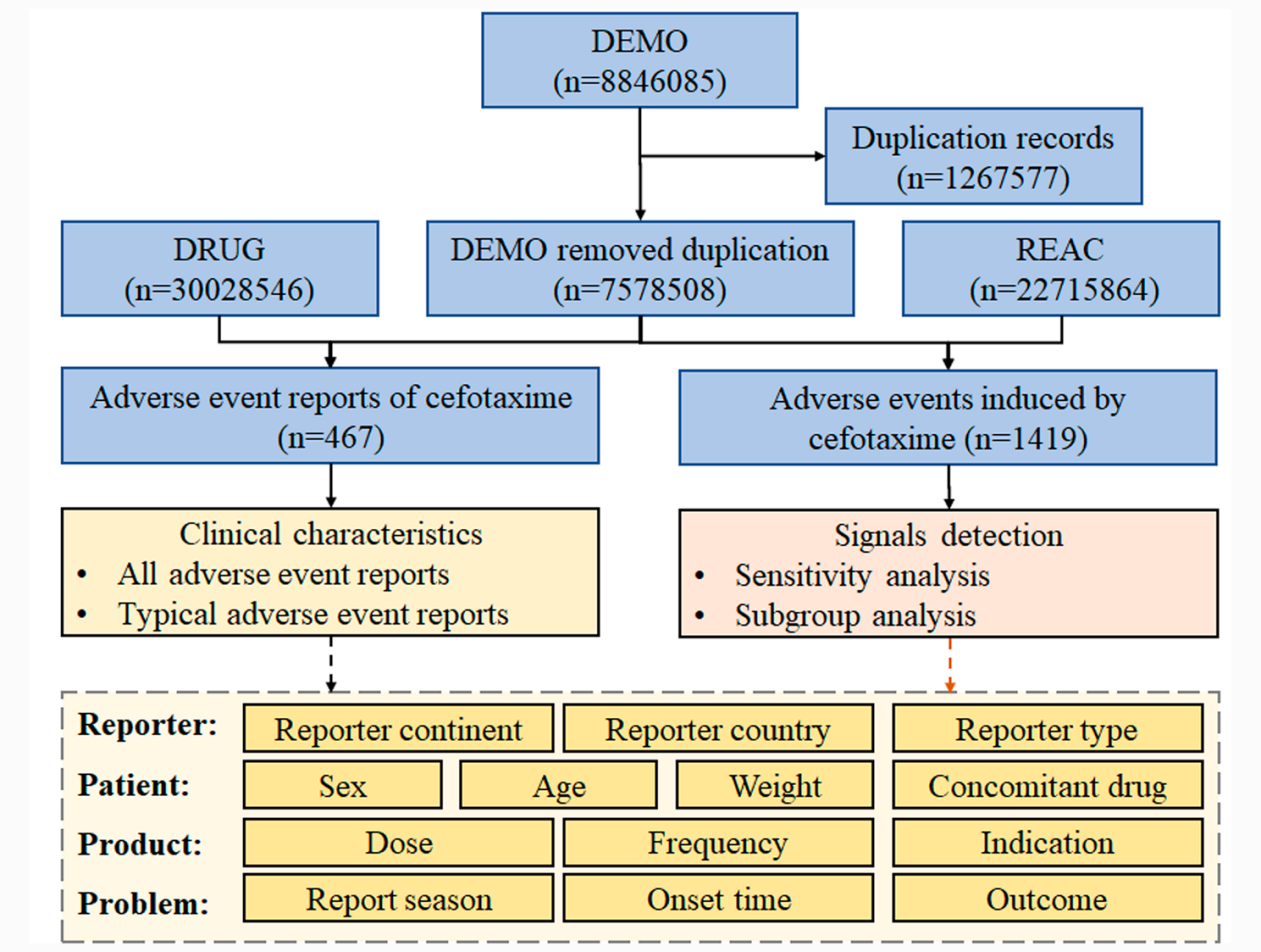
Flow diagram of data collection and analysis of cefotaxime-associated adverse events. Abbreviations: DEMO, patient demographic and administrative information; DRUG, drug information; REAC, coded for the adverse events; PS, primary suspected

A total of 467 adverse event reports and 1419 adverse events associated with cefotaxime were identified. A flow diagram of data collection and analysis of cefotaxime-associated adverse events is shown in Fig. 1.

**Fig. 2:**
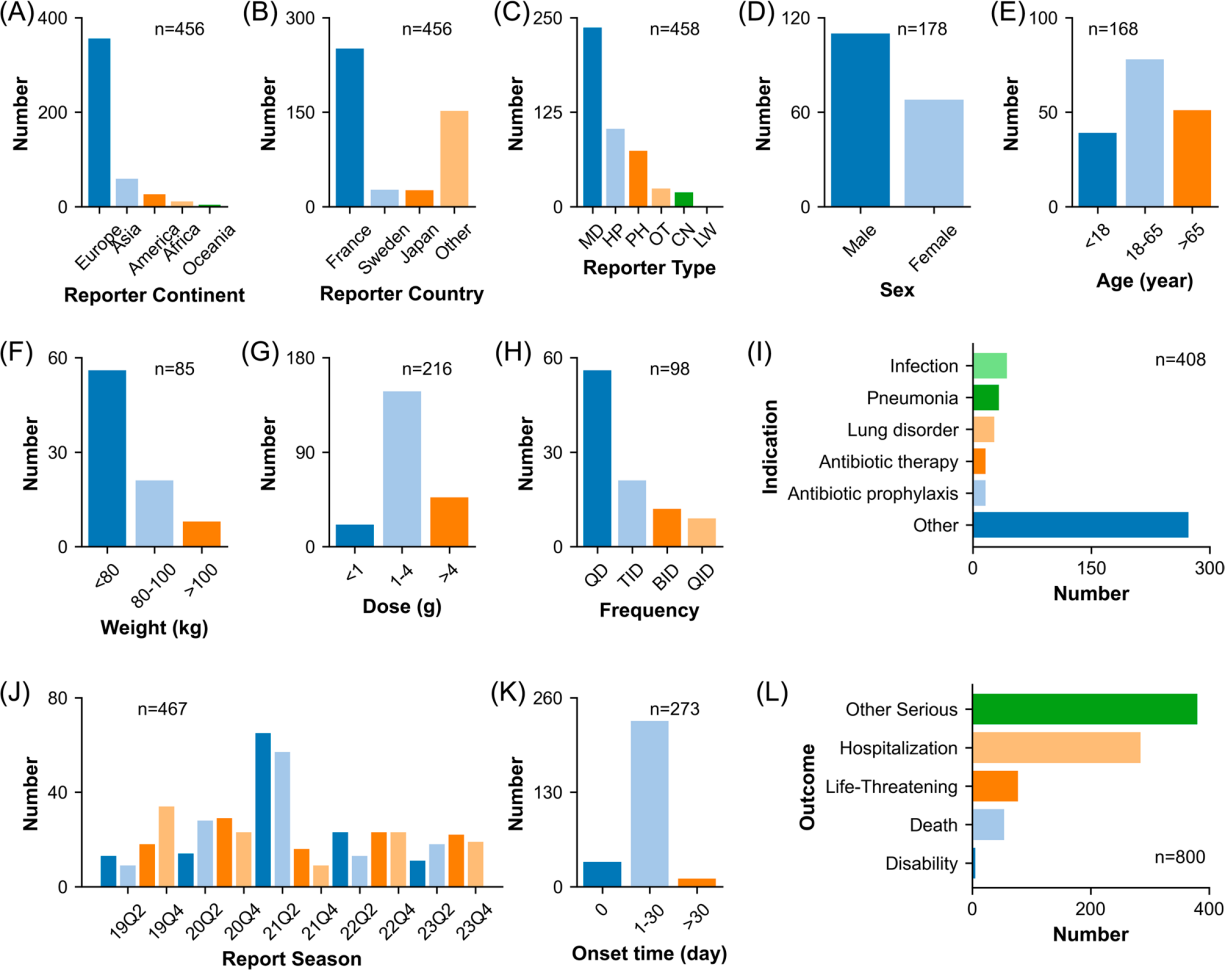
Clinical characteristics of cefotaxime-associated adverse event reports. (**A**) Reporter continent. (**B**) Reporter country. (**C**) Reporter type. (**D**) Sex. (**E**) Age. (**F**) Weight. (**G**) Dose. (**H**) Frequency. (**I**) Indication. (**J**) Report season. (**K**) Onset time. (**L**) Outcome. Abbreviation: MD, Physician; HP, Health-professional; PH, pharmacist; OT, other health-professional; CN, consumer; LW, lawyer; QD, quaque die; TID, ter in die; BID, bis in die; QID, quater in die

The clinical characteristics of the cefotaxime-associated adverse event reports were examined based on valid data, as shown in Fig. 2. Reporters came from 35 countries across 5 continents. The majority of reports came from Europe (*n* = 356), with France being the largest contributor (*n* = 251). Figure 2B illustrates the top three countries based on report numbers, while the complete report numbers for all countries are available in Supplementary Table S5. Physicians filed 237 reports, being the primary reporter type.

In terms of patient, there were more reports for males compared to females (110 versus 68). The majority of patients fell within the 18–65 age range (*n* = 78), with an average age of 45.2 years. Most patients weighed under 80 kg (*n* = 56).

Concerning the product, doses of 1–4 g were the most commonly reported single dose (*n* = 148), and quaque die (QD) was the most common frequency (*n* = 56). The predominant indication was “infection” (*n* = 43).

Regarding the problem, the number of reports ranged from 9 to 65, with an average of 23 reports per quarter. The majority of events occurred within 1–30 days of drug intake (*n* = 228). In this study, the reports with serious clinical outcomes were categorized as 5 categories, including “hospitalization-initial or prolonged”, “life-threatening”, “death”, “disability”, and “other serious”. The most frequent outcome of cefotaxime-associated adverse event reports was “other serious” (*n* = 380).

**Fig. 3:**
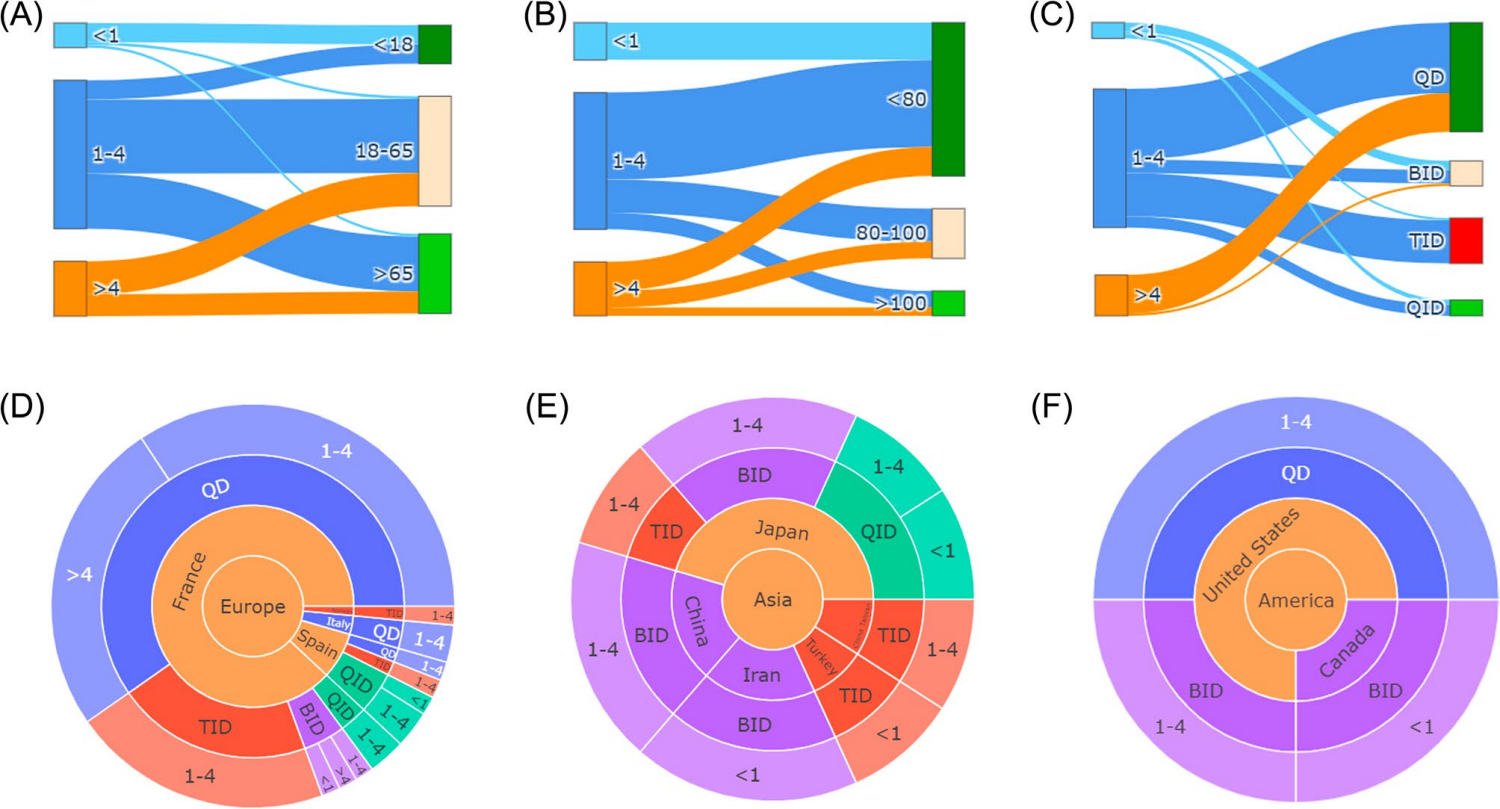
Relationship between dose, age, weight, frequency, and reporter country. (**A**) Sankey plot of dose and age. (**B**) Sankey plot of dose and weight. (**C**) Sankey plot of dose and frequency. (**D**) Sunburst plot of dose and frequency distribution across Europe. (**E**) Sunburst plot of dose and frequency distribution across Asia. (**F**) Sunburst plot of dose and frequency distribution across America. The sunburst plots from the inside out represents the continents, countries, frequencies, and doses. Abbreviation: QD, quaque die; BID, bis in die; TID, ter in die; QID, quater in die

Sankey plots illustrating the relationships among age, weight, frequency, and dose are depicted in Fig. 3A–C. In patients taking doses below 1 g, 77.8% were neonates or children, ranging from 4 days to 7 years old, with an average age of 2.5 years. The remaining 22.2% ranged from 63 to 84 years, with an average age of 72.5 years. Among these patients, the weight ranged from 0.65 to 50.35 kg, with an average weight of 13.9 kg. For cases reported once-daily frequency, all single doses utilized exceeding 1 g. Figure 3D–F depict the sunburst plots illustrating dose and frequency distribution across Europe, Asia, and America (the top three reporter continents in terms of reports number). In comparison to Asia, Europe and America more commonly reported once-daily frequency.

### Signals detection

The number and signal strength of cefotaxime at the SOC level are described in Supplementary Table S6. The significant SOCs that met four criteria were “skin and subcutaneous tissue disorders”, “hepatobiliary disorders” and “blood and lymphatic system disorders”. Among these, “skin and subcutaneous tissue disorders” had the highest number of reports, and “hepatobiliary disorders” exhibited the highest signal strength. At the PT level, the ROR, PRR, BCPNN, and MGPS algorithms detected 156, 97, 87, and 233 signals, respectively. A total of 79 signals conformed to the four algorithms simultaneously. 16 signals, including 13 signals related to the SOC of “infections and infestations”, 2 signals consistent with indications, and 1 signal of “drug resistance”, which may result from disease progression, failure to treatment, or pathogen resistance, were considered as cefotaxime-unrelated signals. The number and signal strength of 16 cefotaxime-unrelated signals at the PT level are listed in Supplementary Table S7. After excluding the 16 cefotaxime-unrelated signals, 63 cefotaxime-associated signals were detected at the PT level. The number and signal strength of 63 cefotaxime-associated signals are shown in Supplementary Table S8. The top three most common cefotaxime-associated signals were all associated with SOC of “skin and subcutaneous tissue disorders” (SOC: 10040785), including “drug reaction with eosinophilia and systemic symptoms”, “rash” and “rash maculo-papular”, which are consistent with the labels of cefotaxime. In terms of ROR, PRR, IC and EBGM values, “umbilical erythema” emerged as the most significant cefotaxime-associated signal. Notably, two new signals related to severe hepatobiliary disorders were identified, including “hepatic failure” and “acute hepatic failure”, which were not highlighted in the cefotaxime label or in previous studies.

### Sensitivity analysis

**Fig. 4:**
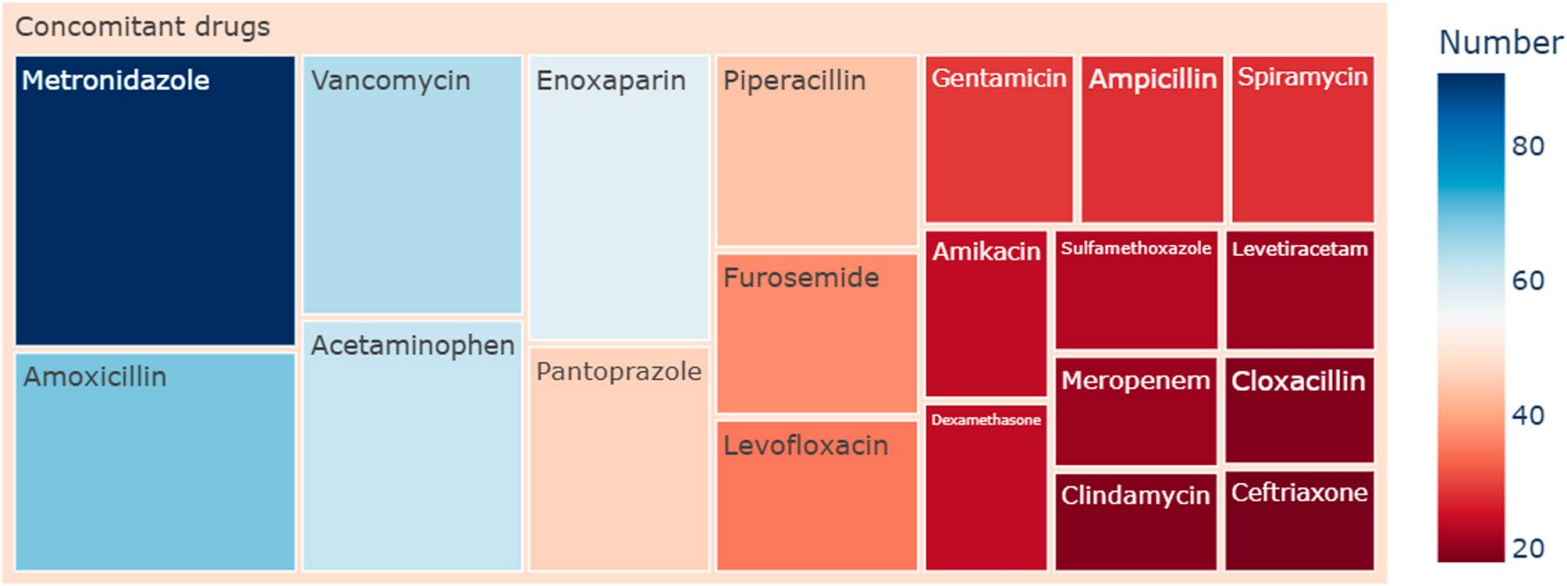
Rectangular tree plot of top 20 ranked concomitant drugs of cefotaxime-associated adverse event reports. The reports of amoxicillin/clavulanate potassium, amoxicillin/clavulanic acid and amoxicillin were merged into the category of amoxicillin. The reports of piperacillin/tazobactam and piperacillin were merged into the category of piperacillin. The reports of ampicillin/sulbactam and ampicillin were merged into the category of ampicillin. The reports of sulfamethoxazole/trimethoprim and sulfamethoxazole were consolidated into the category of sulfamethoxazole

Figure 4 illustrates a rectangular tree plot that displays the occurrence of the top 20 ranked concomitant drugs in the 467 cefotaxime-associated adverse event reports. Among these top 20 drugs, antibiotics made up 14 of them, including metronidazole (*n* = 91), amoxicillin (*n* = 69), vancomycin (*n* = 64), piperacillin (*n* = 44), levofloxacin (*n* = 35), gentamicin (*n* = 29), ampicillin (*n* = 28), spiramycin (*n* = 28), amikacin (*n* = 24), sulfamethoxazole (*n* = 23), meropenem (*n* = 21), clindamycin (*n* = 19), cloxacillin (*n* = 19), and ceftriaxone (*n* = 18). Sensitivity analyses detected 59, 60 and 56 cefotaxime-associated signals at the PT level after excluding reports involving the top three commonly co-administered medications (metronidazole, amoxicillin and vancomycin), as shown in Supplementary Tables S9-S11. In contrast to the main findings, where “umbilical erythema” was identified as a significant adverse event signal for cefotaxime, the sensitivity analysis after excluding reports with vancomycin found it to be non-significant. Sensitivity analysis further verified the significance of two new adverse event signals: “hepatic failure”, and “acute hepatic failure”, after excluding reports with metronidazole, amoxicillin and vancomycin. The clinical characteristics of “hepatic failure” and “acute hepatic failure” reports were further analyzed to explore other potential influencing factors, as shown in Supplementary Figure S1.

### Subgroup analysis

**Fig. 5:**
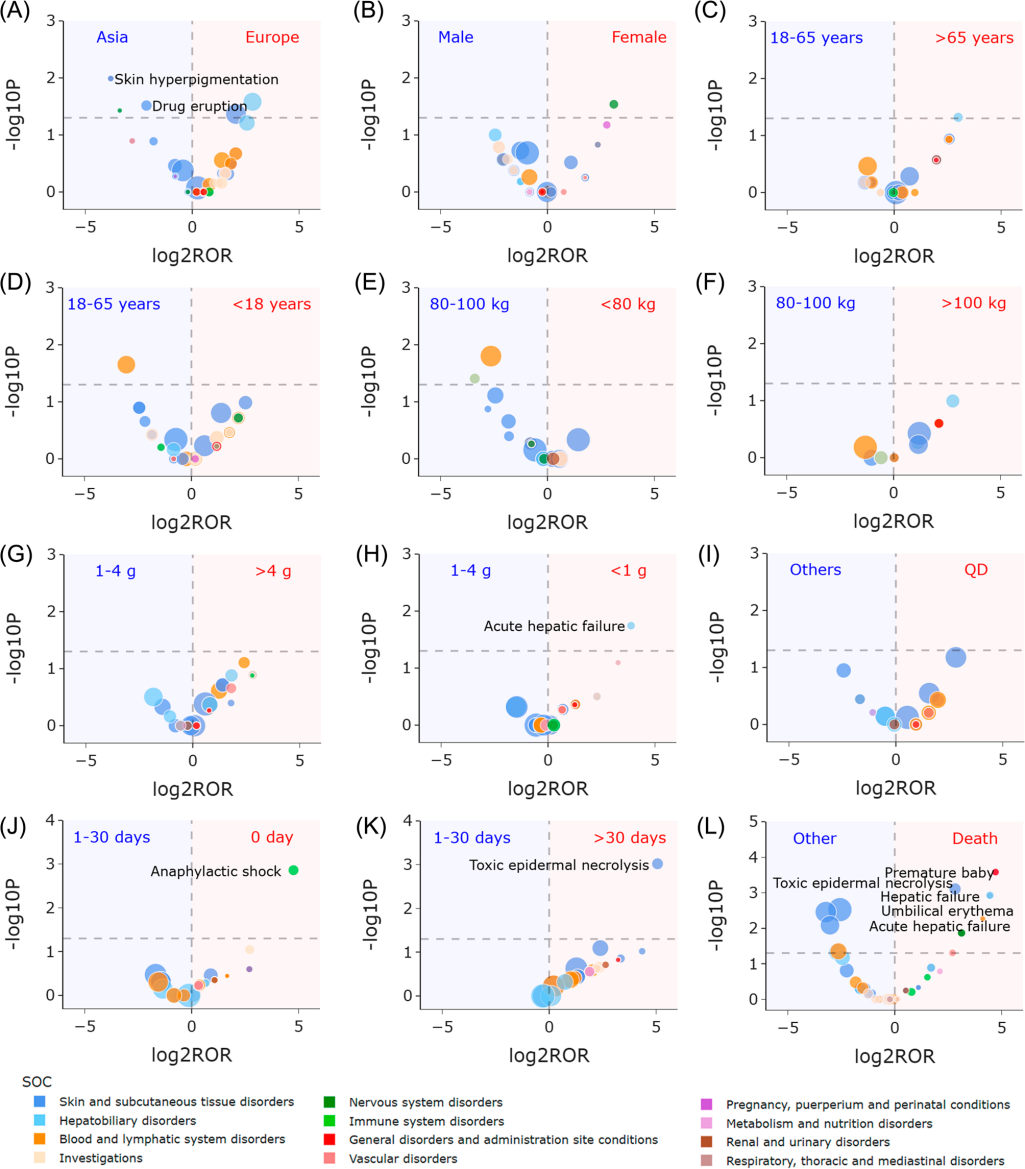
Volcano plots for difference detection of cefotaxime signals. (**A**) Differences between Europe and Asia. (**B**) Difference between females and males. (**C**) Difference between patients with age > 65 years and 18–65 years. (**D**) Difference between patients with age < 18 years and 18–65 years. (**E**) Difference between patients with weight < 80 kg and 80–100 kg. (**F**) Difference between patients with weight > 100 kg and 80–100 kg. (**G**) Difference between patients taking signal dose > 4 g and 1–4 g. (**H**) Difference between patients taking signal dose < 1 g and 1–4 g. (**I**) Difference between patients taking frequency once a day and 2–4 times a day. (**J**) Difference between onset time 0 day and 1–30 days. (**K**) Difference between onset time > 30 days and 1–30 days. (**L**) Difference between patients with “death” outcome and other outcomes. In these volcano plots, the sizes of each point represent the number of reports of each PT induced by cefotaxime. 63 cefotaxime-associated signals are shown. Abbreviation: QD, quaque die

The volcano plots in Fig. 5 depict the subgroup analyses of cefotaxime signals with respect to reporter (reporter continent), patient (sex, age, and weight), product (dose and frequency), and problem (onset time and outcome). Notably, significant variations were observed in relation to reporter continent, dose, onset time, and outcome. Compared to Europe, Asia exhibited a higher reported frequency of “skin and subcutaneous tissue disorders”, especially “skin hyperpigmentation” and “drug eruption” (Europe vs. Asia: “skin hyperpigmentation”, ROR 0.071, 95% CI 0.008–0.641, P 0.010, “drug eruption”, ROR 0.227, 95% CI 0.060–0.851, P 0.031). Patients taking dose less than 1 g were found to have a higher reported frequency of “acute hepatic failure” compared to those taking dose 1–4 g (< 1 g vs. 1–4 g: “acute hepatic failure”, ROR 14.735, 95% CI 1.510-143.801, P 0.018). In terms of onset time, “anaphylactic shock” was more likely to occur on the day of medication, and “toxic epidermal necrolysis” was more common after 30 days compared to the onset time of 1–30 days (0 day vs. 1–30 days: “anaphylactic shock”, ROR 27.152, 95% CI 2.989-246.649, P 0.001; >30 days vs. 1–30 days: “toxic epidermal necrolysis”, ROR 33.525, 95% CI 5.301-212.034, P 0.001). Furthermore, several signals reported more frequently in cases with “death” outcomes, such as “premature baby”, “toxic epidermal necrolysis”, “hepatic failure”, “umbilical erythema”, and “acute hepatic failure” (“death” vs. others: “premature baby”, ROR 26.182, 95% CI 3.139–218.400, *P* < 0.001; “toxic epidermal necrolysis”, ROR 7.052, 95% CI 2.289–21.733, P 0.001; “hepatic failure”, ROR 21.863, 95% CI 2.544-187.926, P 0.001; “umbilical erythema”, ROR 21.736, 95% CI 2.529-186.827, P 0.001; “acute hepatic failure”, ROR 17.323, 95% CI 1.928-155.625, P 0.005).

## Discussion

This study conducted a thorough analysis of the adverse event profiles associated with cefotaxime. The results revealed several notable points.

### Dosing frequency

Regarding frequency, the most prevalent frequency associated with cefotaxime-related adverse event reports was once daily, with all single doses utilized exceeding 1 g. This study revealed that Europe and America reported once-daily dosing more frequently than Asia. Further scrutiny of indications showed that the reports with once-daily frequency were primarily used for treating infections such as “meningitis”, “colitis”, “urinary tract infections”, and “sepsis”. Cefotaxime is a time-dependent antibiotic, meaning the longer the concentration in the body remains above the minimum inhibitory concentration (MIC), the better the antibacterial activity [28]. As per the labels of cefotaxime, the recommended frequency for cefotaxime typically ranges from 2 to 4 times a day. Once-daily dosing regimen not only fails to maintain consistent blood drug levels, impedes antimicrobial efficacy, increases the risk of adverse events, and may also potentially accelerate the emergence of resistant pathogens. Till now, the emergence of resistant pathogens has become a new global public health threat [29]. In recent years, the resistance rate of Gram-negative bacteria to third-generation cephalosporins continues to increase, especially Escherichia coli [30–34]. Cefotaxime-resistant Escherichia coli have been isolated from cow milk [35], floor [36], and waters [37]. This study reveals the existence of non-recommended frequency in Europe and America, which warrant clinical attention when using cefotaxime. It is important to note that this study only included patients who experienced adverse events from the FAERS database and could not assess the dosing frequency of all patients treated with cefotaxime.

### Concomitant drugs

In this study, cefotaxime was often used in combination with various antibiotics. Despite its broad-spectrum bactericidal activity, cefotaxime shows limited efficacy against Pseudomonas aeruginosa infections and is not recommended as a sole therapy [1]. The most commonly reported combinations of cefotaxime were metronidazole, amoxicillin and vancomycin. Due to the complementary antibacterial spectra of cefotaxime and metronidazole, their combination is frequently recommended in clinical practice. Soriano et al. demonstrated that while anaerobic bacteria degrade cefotaxime, the inclusion of metronidazole effectively eradicates the anaerobes and safeguards cefotaxime from degradation, enabling it to eliminate Escherichia coli [38]. However, metronidazole may cause adverse reactions such as nervous system disorders and gastrointestinal disorders. Therefore, enhanced monitoring for these effects is recommended when co-administering cefotaxime with metronidazole. Cefotaxime and amoxicillin are both β-lactam antibiotics. In addition to amoxicillin, β-lactam antibiotics including piperacillin, ampicillin, and ceftriaxone were also identified in the top 20 combined medications. The use of antibiotics with similar mechanisms of action in combination may not provide benefits and could increase the risk of adverse events, warranting clinical attention. Moreover, concurrent administration of cefotaxime with drugs such as vancomycin, gentamicin, and amikacin may increase the risk of renal toxicity, necessitating intensive monitoring of renal function [1]. Studies have shown that the combination drugs like sulbactam [39], delafloxacin [40], fucoxanthin [41], etimicin sulfate [42], Tunisian Thymus capitatus essential oil [43], corosolic acid [44], chlorogenic acid [45], and eugenol [46] can enhance the therapeutic effects of cefotaxime. Therefore, the utilization of appropriate combination drugs needs special attention in the clinical use of cefotaxime.

### Hepatobiliary disorders

Utilizing four disproportionality algorithms, the most significant SOC identified was “hepatobiliary disorders”. This study observed “hepatocellular injury”, “hepatic cytolysis” and “cholestasis” as the most frequently adverse reactions related to hepatobiliary disorders, which have been reported in previous literatures [47, 48]. Additionally, this study uncovered two severe hepatobiliary disorders: “hepatic failure” and “acute hepatic failure”. The sensitivity analysis further revealed that these two signals remained significant after excluding reports involving commonly co-administered medications. According to the labels for cefotaxime, hepatobiliary related disorders, such as transient liver enzyme elevations, are only mentioned among the less common adverse reactions (occurring in < 1% of cases), and no severe hepatobiliary disorders have been documented. The reports with “hepatic failure” or “acute hepatic failure” were only reported in nine cases, potentially explaining why they were not emphasized in cefotaxime labels or previous studies.

Generally, higher doses of medication are more likely to cause severe hepatobiliary disorders. However, subgroup analysis revealed an unexpected finding: a higher frequency of “acute hepatic failure” in reports with doses below 1 g, which has raised our concerns. Clinical characteristics analysis revealed that patients experiencing “hepatic failure” or “acute hepatic failure” were either neonates, children or underweight elderly individuals. Importantly, eight out of the nine cases resulted in death, highlighting the seriousness of these severe hepatobiliary disorders. Although cefotaxime is commonly used to treat bacterial infections in neonates, children, and the elderly [49, 50], there is considerable variability in cefotaxime concentrations among patients of different ages, particularly in neonates [51]. Boehm et al. demonstrated that infants exhibit limited monooxygenase system capacity of the liver during the first weeks of life, along with a specific, reversible impact of cefotaxime on the hepatocellular system [47]. This study reveals that severe hepatobiliary disorders were observed in neonates, children, or underweight elderly patients even when using adjusted doses below 1 g. While the dose-response relationship still need be validated through real-world cohort studies in the future, personalized dosing regimens for cefotaxime based on age-appropriate pharmacokinetic data have been shown to be safe and effective in the young [52]. For underweight elderly patients, dosing based on ideal body weight (IBW), total body weight (TBW), or adjusted body weight (AdjBW) could help reduce the risk of adverse events [53, 54]. These findings highlight the necessity of personalized dosing strategies and intensified liver function monitoring in neonates, children, and elderly patients with lower body weight. Given the limited number of reports, this study suggests further investigations to confirm the correlation of these two severe signals with cefotaxime.

### Umbilical erythema

Among the 63 cefotaxime-associated signals, the most significant signal identified was “umbilical erythema”, a symptom not previously documented in the cefotaxime labels. Despite there being only four reports, the notably higher signal strength of “umbilical erythema” warrants discussion. Umbilical erythema is considered to be an infectious disease typical of neonates, which is primarily caused by omphalitis, infectious anomalies of the ureter, and infectious abnormalities of the umbilical gastrointestinal tract [55, 56]. It may be considered a reliable sign of potential necrotizing enterocolitis and bacterial peritonitis, which can progress to necrotizing fasciitis, with high morbidity and mortality rates [55, 57]. Since data mining only revealed the concurrence of cefotaxime use and “umbilical erythema”, this study further examined the reactions of these four cases. All four cases, in addition to “umbilical erythema”, simultaneously reported reactions including “premature baby”, “fetal exposure during pregnancy”, “renal failure”, “respiratory disorder”, “hypotonia”, “bacterial pneumonia”, “sepsis”, and “Stenotrophomonas infection”. The sensitivity analysis revealed that the association became non-significant after excluding reports involving vancomycin, a commonly co-administered drug. As a glycopeptide antibiotic, vancomycin is widely used to treat severe infections [58, 59], and its combination with β-lactams has shown synergistic antibacterial effects against methicillin-resistant Staphylococcus aureus bacteremia [60]. These findings suggest that the observed umbilical erythema may be attributable to the underlying infection or concomitant medications rather than cefotaxime alone. The causal relationship between cefotaxime and umbilical erythema remains uncertain and requires further investigation. Notably, all cases resulted in “death” outcome. Given that umbilical erythema can be associated with serious clinical outcomes, this study recommends maintaining a high index of suspicion when administering cefotaxime to premature baby-particularly for conditions such as bacterial pneumonia, sepsis, and Stenotrophomonas infections.

### Subgroup analysis across continents

The study identified significant disparities in adverse event signals related to cefotaxime among different reporter continents. Genetic polymorphisms are frequently observed across various races, affecting protein expression, drug absorption/metabolism functions, and in vivo drug exposure, leading to variations in drug responses [61]. It is estimated that over half of all adverse drug reactions can be attributed to the expression of polymorphic genes [62]. In the liver, cefotaxime transforms into desacetylcefotaxime [1]. The distribution of common variant alleles of human drug-metabolizing cytochrome P450 genes varies among different ethnic populations, potentially leading to differences in cefotaxime metabolism [63]. Furthermore, this study revealed different medical practices across continents, especially in dosing frequency, which may influence the adverse events related to cefotaxime. Alden et al. found that Filipinos have lower levels of antibiotic knowledge, express higher perceived need, and report more frequent use; whites are at the opposite end on all of these measures [64]. The differences in adverse events may also be influenced by environmental exposures [65] and reporting practices among continents. Although the factors influencing the variations between continents are complex, these results suggest focused monitoring strategies for populations from different continents.

### Subgroup analysis across onset times

Although the majority of events occurred within 1–30 days, this study highlighted different patterns in the onset times of acute and delayed adverse events. “Anaphylactic shock” occurrences on the day of medication were notably higher, emphasizing the need for immediate monitoring post-medication administration. Anaphylaxis is a severe hypersensitivity reaction with the potential for fatality, and was disproportionally reported for drugs like cephalosporins, penicillins, quinolones, and glycopeptides [66]. Recently, the prevalence of anaphylaxis is increasing and the number of cases of fatal anaphylaxis appears to be rising [67]. Patients with anaphylaxis are manageable with epinephrine, antihistamines, vasopressors, or corticosteroids [1].

In addition to acute adverse events, long-term adverse events with cefotaxime are also easily overlooked. This study revealed that “toxic epidermal necrolysis”, a potentially fatal adverse event, was more commonly observed in patients using cefotaxime for over 30 days. The symptoms can improve rapidly through swift and decisive medical interventions, such as the infusion of high doses of immunoglobulin. Paquet et al. documented a case of toxic epidermal necrolysis (involving 40% of the body surface) in a 75-year-old woman following cefotaxime administration [68]. Subsequent use of meropenem triggered an immediate recurrence of toxic epidermal necrolysis, spreading to previously unaffected skin areas with fatal consequences [68]. The study revealed the cross-reactivity between cefotaxime and meropenem [68]. Therefore, patients experiencing toxic epidermal necrolysis due to medications containing the β-lactam ring usage should use cefotaxime with caution. Long-term strategies such as managing risk factors and providing education on avoidance are essential for minimizing the risk of recurrence of these severe adverse events. The study underscores the importance of promptly and effectively addressing severe adverse events such as “anaphylactic shock” and “toxic epidermal necrolysis” at different periods of cefotaxime use.

### Strengths and limitations

This study has several strengths. Firstly, the study systematically analyzed the clinical characteristics of adverse event reports related to cefotaxime, identifying issues such as dosing frequency and concurrent medication use. Secondly, the research uncovered two new severe hepatobiliary disorders, and further explored the characteristics associated with them. Thirdly, the study identified significant differences in cefotaxime adverse events concerning the reporter continent, dose, onset time, and outcome, suggesting focused monitoring strategies. To the best of our knowledge, this is the first comprehensive and systematic pharmacovigilance study of cefotaxime concerning variations in adverse event profiles across subgroups. It is hoped that this study will provide a more comprehensive and valuable reference for the adverse event profile of cefotaxime.

There are several noteworthy limitations to be addressed. Firstly, the spontaneous reporting nature of FAERS database may lead to selection bias and incomplete reporting (such as more likely reporting of serious adverse events), which may limit comprehensive adverse event characterization. Secondly, the absence of the total number of cefotaxime users hampers the calculation of adverse event incidence rates. Thirdly, concurrent medications that could either increase or decrease adverse events may influence the results. Although this study tried to reduce the bias induced by concurrent medications through sensitivity analysis, the inherent limitations of the FAERS database constrained our ability to comprehensively address this issue. Moreover, traditional Chinese medicine is known for its antibacterial and hepatoprotective activities [69–73]. However, this study did not take into account the influence of these concurrent medications. Fourthly, renal dysfunction can significantly elevate plasma concentrations [74] and a personalized cefotaxime administration regimen based on renal function is crucial [75, 76]. Nevertheless, this study lacks pertinent information reflecting renal function to assess the appropriateness of cefotaxime dosages. Fifthly, to address potential biases, recent studies for signal detection have increasingly simultaneous used of multiple disproportionality analyses algorithms, which are broadly classified into two main groups: frequency count methods, including ROR, PRR, and the Medicines and Healthcare Products Regulatory Agency (MHRA), and Bayesian methods, such as BCPNN and MGPS algorithms [27]. Nevertheless, it is important to note that the possibility of false positives arising from multiple testing increased [77]. Although methods such as the Bonferroni correction and other sophisticated algorithms can reduce the likelihood of false-positive signals, their application may also increase the risk of overlooking weaker yet clinically significant signals. Moreover, several unresolved issues remain, such as the arbitrary selection of thresholds and the lack of justification for how these thresholds reflect appropriate correction for multiple testing [77]. To date, there is still no established gold standard for handling data from the FAERS database. Therefore, this study adheres to the widely recognized disproportionality analysis methods, employing ROR, PRR, BCPNN, and MGPS to ensure clarity and consistency [78]. However, there is a need to clarify these associations and underlying molecular mechanisms through further studies, such as epidemiological research and bioinformatic analyses [79]. Sixthly, the choice of a comparator holds significant importance in disproportionality analysis [80]. Drawing insights from existing literature on FAERS database management, this study compared the reporting rates of cefotaxime with rates across all other drugs. Moving forward, it is imperative to carefully select more comparators for further exploration of adverse events linked with cefotaxime in future research endeavors. Despite these limitations, leveraging the FAERS database offers invaluable pharmacovigilance data.

## Conclusions

This study conducted post-marketing drug safety monitoring of cefotaxime, offering valuable insights for its clinical management. The findings reveal the existence of once-daily frequency in Europe and America, which warrant clinical attention when using cefotaxime. The identification of two new severe hepatobiliary disorders highlights the critical need for personalized dosing strategies and intensified liver function monitoring for neonates, children, and elderly individuals with lower body weights. Furthermore, the differences in adverse event signals across continent, dose, onset time, and outcome subgroups underscore the necessity of developing targeted monitoring protocols.

## Electronic supplementary material

Below is the link to the electronic supplementary material.

Supplementary Material 1

## Data Availability

Raw data for this article can be downloaded at https://fis.fda.gov/extensions/FPD-QDE-FAERS/FPD-QDE-FAERS.html; further inquiries can be directed to the corresponding author.

## Abbreviations

FDA: Food and Drug Administration
CNS: Central nervous system
SCAR: Severe cutaneous adverse reactions
DRESS: Drug reaction with eosinophilia and systemic symptom
FAERS: Food and Drug Administration Adverse Event Reporting System
PS: Primary suspected
SS: Secondary suspect
C: Concomitant
I: Interacting
PT: Preferred terms
SOC: System organ class
MedDRA: Medical Dictionary for Regulatory Activities
ROR: Reporting odds ratio
PRR: Proportional reporting ratio
BCPNN: Bayesian confidence propagation neural network
MGPS: Multi-item gamma Poisson shrinker
MIC: Minimum inhibitory concentration
IBW: Ideal body weight
TBW: Total body weight
AdjBW: Adjusted body weight
RBC: Red blood cell
HDN: Hemolytic disease of the newborn
