# Supplementary material for: Exploration of adverse event profiles for cefotaxime: a disproportionality analysis using the FDA adverse event reporting system
Source: BMC Pharmacol Toxicol. 2025 Jul 1;26:129. doi: 10.1186/s40360-025-00960-w (PMC12220385; doi:10.1186/s40360-025-00960-w)
Supplement: Supplementary file 1 — Supplementary Material 1 [file 40360_2025_960_MOESM1_ESM.docx]

Supplementary Material

**Supplementary Table S1**. Fourfold table of disproportionality analysis for cefotaxime signal detection.

|  | Target adverse event | Non-target adverse events | Total |
| --- | --- | --- | --- |
| Cefotaxime | a | b | a+b |
| Other drugs | c | d | c+d |
| Total | a+c | b+d | a+b+c+d |

**Supplementary Table S2.** Equations and criteria of four algorithms for cefotaxime signal detection.

| Algorithms | Equation | Criteria |
| --- | --- | --- |
| ROR | ROR=(ad)/(bc) | Lower limit of 95% CI>1, N≥2 |
|  | Lower limit of 95% CI=e^ln(ROR)-1.96(1/a+1/b+1/c+1/d)^0.5^  Upper limit of 95% CI=e^ln(ROR)+1.96(1/a+1/b+1/c+1/d)^0.5^ |  |
| PRR | PRR=a(c+d)/c/(a+b) | PRR≥2, χ2≥4, N≥3 |
|  | χ2=[(ad-bc)^2](a+b+c+d)/[(a+b)(c+d)(a+c)(b+d)] |  |
| BCPNN | IC=log_2_a(a+b+c+d)/(a+c)/(a+b) | IC025>0 |
|  | IC025=E(IC)-2V(IC)^0.5 |  |
| MGPS | EBGM=a(a+b+c+d)/(a+c)/(a+b) | EBGM05>2 |
|  | EBGM05=e^ln(EBGM)-1.96(1/a+1/b+1/c+1/d)^0.5^ |  |

**Abbreviation:** ROR, reporting odds ratio; CI, confidence interval; N, number of co-occurrences; PRR, proportional reporting ratio; χ2, chi-squared; BCPNN, Bayesian confidence propagation neural network; IC, information component; IC025, lower limit of 95% confidence interval of IC; E(IC), IC expectation; V(IC), variance of IC; MGPS, multi-item gamma Poisson shrinker; EBGM, empirical Bayesian geometric mean; EBGM05, lower limit of 95% confidence interval of EBGM.

**Supplementary Table S3**. Fourfold table of disproportionality analysis for subgroup analyses of cefotaxime signals.

|  | Target adverse event with cefotaxime | Non-target adverse events with cefotaxime | Total |
| --- | --- | --- | --- |
| Factor 1 | A | b | a+b |
| Factor 2 | C | d | c+d |
| Total | a+c | b+d | a+b+c+d |

**Supplementary Table S4**. Criteria of ROR and Fisher’s exact test for subgroup analyses of cefotaxime signals.

|  | ROR | Fisher’s exact test |
| --- | --- | --- |
| Signals that factor 1 more frequently to report | ROR>1, Lower limit of 95% CI>1,  N≥2 | P<0.05 |
| Signals that factor 2 more frequently to report | ROR<1, Upper limit of 95% CI<1,  N≥2 | P<0.05 |

**Abbreviation:** ROR, reporting odds ratio; 95% CI, 95% confidence interval; N, number of co-occurrences.

**Supplementary Table S5.** Number of cefotaxime-associated adverse event reports from 35 reporter countries.

| **Reporter country** | **Number** | **Reporter country** | **Number** | **Reporter country** | **Number** |
| --- | --- | --- | --- | --- | --- |
| France | 251 | Iran | 4 | Norway | 2 |
| Sweden | 27 | Russia | 4 | New Aealand | 2 |
| Japan | 26 | Turkey | 4 | Netherlands | 2 |
| Spain | 25 | South Korea | 4 | Pakistan | 1 |
| Canada | 14 | Slovakia | 3 | Hungary | 1 |
| India | 13 | United Kingdom | 3 | Czech Republic | 1 |
| Italy | 13 | Portugal | 2 | China Taiwan | 1 |
| America | 12 | Poland | 2 | Bulgaria | 1 |
| Germany | 9 | Australia | 2 | Belarus | 1 |
| Tunisia | 7 | Belgium | 2 | Finland | 1 |
| Greece | 6 | Egypt | 2 | Iraq | 1 |
| China | 5 | Algeria | 2 |  |  |

**Supplementary Table S6**. Number and signal strength of cefotaxime at the SOC level.

| **SOC** | **Number** | **ROR (95% CI)** | **PRR (χ2)** | **IC (IC025)** | **EBGM (EBGM05)** |
| --- | --- | --- | --- | --- | --- |
| Skin and subcutaneous tissue disorders (SOC: 10040785) ^abcd^ | 331 | 4.92 (4.35-5.57) | 4.01 (792.68) | 2.00 (1.81) | 4.01 (3.54) |
| General disorders and administration site conditions (SOC: 10018065) | 148 | 0.55 (0.46-0.65) | 0.59 (49.98) | -0.75 (-1.00) | 0.59 (0.50) |
| Infections and infestations (SOC: 10021881) ^ac^ | 132 | 1.75 (1.47-2.10) | 1.68 (38.79) | 0.75 (0.48) | 1.68 (1.41) |
| Hepatobiliary disorders (SOC: 10019805) ^abcd^ | 118 | 11.03 (9.14-13.32) | 10.20 (986.45) | 3.35 (2.97) | 10.19 (8.44) |
| Investigations (SOC: 10022891) ^ac^ | 117 | 1.47 (1.22-1.77) | 1.43 (16.05) | 0.52 (0.23) | 1.43 (1.18) |
| Blood and lymphatic system disorders (SOC: 10005329) ^abcd^ | 115 | 5.13 (4.24-6.21) | 4.79 (351.06) | 2.26 (1.93) | 4.79 (3.96) |
| Gastrointestinal disorders (SOC: 10017947) | 89 | 0.79 (0.64-0.98) | 0.80 (4.68) | -0.32 (-0.63) | 0.80 (0.65) |
| Nervous system disorders (SOC: 10029205) | 84 | 0.79 (0.64-0.99) | 0.81 (4.26) | -0.31 (-0.63) | 0.81 (0.65) |
| Respiratory, thoracic and mediastinal disorders (SOC: 10038738) | 50 | 0.77 (0.58-1.03) | 0.78 (3.20) | -0.36 (-0.76) | 0.78 (0.59) |
| Injury, poisoning and procedural complications (SOC: 10022117) | 48 | 0.26 (0.19-0.34) | 0.28 (99.49) | -1.82 (-2.22) | 0.28 (0.21) |
| Renal and urinary disorders (SOC: 10038359) ^ac^ | 43 | 1.48 (1.09-2.01) | 1.47 (6.55) | 0.55 (0.10) | 1.47 (1.08) |
| Immune system disorders (SOC: 10021428) ^ac^ | 31 | 1.81 (1.27-2.59) | 1.80 (11.06) | 0.84 (0.29) | 1.80 (1.26) |
| Vascular disorders (SOC: 10047065) | 29 | 1.10 (0.76-1.59) | 1.10 (0.25) | 0.13 (-0.41) | 1.10 (0.76) |
| Psychiatric disorders (SOC: 10037175) | 20 | 0.25 (0.16-0.39) | 0.26 (44.46) | -1.94 (-2.53) | 0.26 (0.17) |
| Metabolism and nutrition disorders (SOC: 10027433) | 20 | 0.73 (0.47-1.13) | 0.73 (2.04) | -0.45 (-1.07) | 0.73 (0.47) |
| Musculoskeletal and connective tissue disorders (SOC: 10028395) | 16 | 0.21 (0.13-0.35) | 0.22 (45.90) | -2.17 (-2.81) | 0.22 (0.14) |
| Cardiac disorders (SOC: 10007541) | 15 | 0.53 (0.32-0.88) | 0.54 (6.18) | -0.90 (-1.59) | 0.54 (0.32) |
| Pregnancy, puerperium and perinatal conditions (SOC: 10036585) | 6 | 1.16 (0.52-2.58) | 1.16 (0.13) | 0.21 (-0.91) | 1.16 (0.52) |
| Reproductive system and breast disorders (SOC: 10038604) | 3 | 0.34 (0.11-1.05) | 0.34 (3.86) | -1.56 (-2.74) | 0.34 (0.11) |
| Ear and labyrinth disorders (SOC: 10013993) | 2 | 0.34 (0.09-1.37) | 0.34 (2.52) | -1.54 (-2.86) | 0.34 (0.09) |
| Congenital, familial and genetic disorders (SOC: 10010331) | 1 | 0.26 (0.04-1.85) | 0.26 (2.10) | -1.94 (-3.32) | 0.26 (0.04) |
| Eye disorders (SOC: 10015919) | 1 | 0.04 (0.01-0.26) | 0.04 (25.44) | -4.75 (-5.85) | 0.04 (0.01) |

^a^: SOCs met the criteria of ROR algorithm; ^b^: SOCs met the criteria of PRR algorithm; ^c^: SOCs met the criteria of BCPNN algorithm; ^d^: SOCs met the criteria of MGPS algorithm. SOC, system organ class; **Abbreviation:** ROR, reporting odds ratio; CI, confidence interval; PRR, proportional reporting ratio; χ2, chi-squared; IC, information component; IC025, lower limit of 95% confidence interval of IC; EBGM, empirical Bayesian geometric mean; EBGM05, lower limit of 95% confidence interval of EBGM.

**Supplementary Table S7.** Number and signal strength of 16 cefotaxime-unrelated signals at the PT level.

| **PT** | **Number** | **ROR (95% CI)** | **PRR (χ2)** | **IC (IC025)** | **EBGM (EBGM05)** |  |
| --- | --- | --- | --- | --- | --- | --- |
| **Infections and infestations (SOC: 10021881)** | | | | | | |
| Sepsis (PT: 10040047) | 9 | 3.88 (2.01-7.47) | 3.86 (19.11) | 1.95 (0.67) | 3.86 (2.00) |  |
| Candida infection (PT: 10074170) | 8 | 17.78 (8.87-35.64) | 17.69 (125.84) | 4.14 (1.67) | 17.67 (8.81) |  |
| Septic shock (PT: 10040070) | 6 | 6.44 (2.89-14.37) | 6.42 (27.46) | 2.68 (0.76) | 6.42 (2.88) |  |
| Pneumonia bacterial (PT: 10060946) | 5 | 20.64 (8.57-49.70) | 20.58 (93.01) | 4.36 (1.09) | 20.55 (8.54) |  |
| Rash pustular (PT: 10037888) | 5 | 47.78 (19.83-115.12) | 47.61 (227.51) | 5.57 (1.26) | 47.47 (19.70) |  |
| Clostridium difficile colitis (PT: 10009657) | 4 | 18.07 (6.77-48.25) | 18.03 (64.26) | 4.17 (0.74) | 18.01 (6.75) |  |
| Bacterial infection (PT: 10060945) | 4 | 9.64 (3.61-25.72) | 9.61 (30.86) | 3.26 (0.53) | 9.61 (3.60) |  |
| Stenotrophomonas infection (PT: 10054138) | 4 | 112.25 (41.93-300.53) | 111.94 (436.75) | 6.80 (0.97) | 111.17 (41.52) |  |
| Pseudomembranous colitis (PT: 10037128) | 3 | 91.84 (29.49-286.02) | 91.65 (267.44) | 6.51 (0.50) | 91.13 (29.26) |  |
| Sepsis neonatal (PT: 10040049) | 3 | 212.00 (67.78-663.03) | 211.55 (620.47) | 7.71 (0.52) | 208.80 (66.76) |  |
| Abscess (PT: 10000269) | 3 | 8.18 (2.63-25.39) | 8.16 (18.85) | 3.03 (0.10) | 8.16 (2.63) |  |
| Device related infection (PT: 10064687) | 3 | 10.95 (3.52-33.99) | 10.92 (27.03) | 3.45 (0.20) | 10.92 (3.52) |  |
| Pathogen resistance (PT: 10034133) | 3 | 9.79 (3.15-30.41) | 9.77 (23.62) | 3.29 (0.17) | 9.77 (3.15) |  |
| **Consistent with indications** | | | | | | |
| Pyrexia (PT: 10037660) | 38 | 5.19 (3.76-7.17) | 5.08 (125.14) | 2.34 (1.73) | 5.08 (3.68) |  |
| Respiratory disorder (PT: 10038683) | 4 | 6.34 (2.37-16.91) | 6.32 (17.92) | 2.66 (0.32) | 6.32 (2.37) |  |
| **Others** | | | | | | |
| Drug resistance (PT: 10059866) | 4 | 5.56 (2.09-14.85) | 5.55 (14.93) | 2.47 (0.25) | 5.55 (2.08) |  |

**Abbreviation:** PT, preferred term; ROR, reporting odds ratio; CI, confidence interval; PRR, proportional reporting ratio; χ2, chi-squared; IC, information component; IC025, lower limit of 95% confidence interval of IC; EBGM, empirical Bayesian geometric mean; EBGM05, lower limit of 95% confidence interval of EBGM.

**Supplementary Table S8**. Number and signal strength of 63 cefotaxime-associated signals at the PT level.

| **PT** | **Number** | **ROR (95% CI)** | **PRR (χ2)** | **IC (IC025)** | **EBGM (EBGM05)** |  |
| --- | --- | --- | --- | --- | --- | --- |
| **Skin and subcutaneous tissue disorders (SOC: 10040785)** | | | | | | |
| Drug reaction with eosinophilia and systemic symptoms (PT: 10073508) | 51 | 78.05 (58.98-103.29) | 75.28 (3722.43) | 6.23 (4.54) | 74.94 (56.63) |  |
| Rash (PT: 10037844) | 40 | 4.00 (2.92-5.48) | 3.92 (87.61) | 1.97 (1.41) | 3.92 (2.86) |  |
| Rash maculo-papular (PT: 10037868) | 33 | 69.37 (49.09-98.05) | 67.78 (2162.96) | 6.08 (4.01) | 67.50 (47.76) |  |
| Erythema (PT: 10015150) | 19 | 3.61 (2.29-5.67) | 3.57 (35.34) | 1.84 (1.01) | 3.57 (2.27) |  |
| Toxic epidermal necrolysis (PT: 10044223) | 13 | 43.94 (25.43-75.91) | 43.54 (539.02) | 5.44 (2.65) | 43.43 (25.14) |  |
| Purpura (PT: 10037549) | 12 | 77.39 (43.78-136.78) | 76.74 (892.89) | 6.26 (2.68) | 76.38 (43.21) |  |
| Acute generalised exanthematous pustulosis (PT: 10048799) | 12 | 63.97 (36.20-113.04) | 63.44 (734.62) | 5.98 (2.64) | 63.19 (35.76) |  |
| Toxic skin eruption (PT: 10057970) | 11 | 54.28 (29.96-98.33) | 53.86 (568.87) | 5.75 (2.48) | 53.69 (29.63) |  |
| Dermatitis exfoliative generalised (PT: 10012456) | 10 | 83.22 (44.61-155.26) | 82.64 (802.45) | 6.36 (2.42) | 82.22 (44.07) |  |
| Drug eruption (PT: 10013687) | 9 | 23.78 (12.34-45.82) | 23.64 (194.88) | 4.56 (1.94) | 23.60 (12.25) |  |
| Rash erythematous (PT: 10037855) | 8 | 8.60 (4.29-17.22) | 8.55 (53.36) | 3.10 (1.25) | 8.55 (4.27) |  |
| Stevens-Johnson syndrome (PT: 10042033) | 7 | 21.25 (10.11-44.68) | 21.15 (134.23) | 4.40 (1.56) | 21.12 (10.05) |  |
| Skin reaction (PT: 10040914) | 5 | 16.57 (6.88-39.90) | 16.52 (72.84) | 4.04 (1.02) | 16.50 (6.86) |  |
| Petechiae (PT: 10034754) | 5 | 24.08 (10.00-57.98) | 24.00 (110.05) | 4.58 (1.13) | 23.96 (9.95) |  |
| Cutaneous vasculitis (PT: 10011686) | 4 | 43.27 (16.19-115.59) | 43.15 (164.25) | 5.43 (0.90) | 43.03 (16.11) |  |
| Umbilical erythema (PT: 10055029) | 4 | 3777.08 (1269.34-11239.19) | 3766.44 (12189.62) | 11.57 (0.89) | 3049.21 (1024.73) |  |
| Rash morbilliform (PT: 10037870) | 4 | 68.45 (25.60-183.02) | 68.26 (263.99) | 6.09 (0.94) | 67.98 (25.42) |  |
| Dermatitis bullous (PT: 10012441) | 4 | 28.12 (10.53-75.10) | 28.05 (104.16) | 4.81 (0.84) | 28.00 (10.48) |  |
| Skin lesion (PT: 10040882) | 4 | 6.16 (2.31-16.43) | 6.14 (17.22) | 2.62 (0.30) | 6.14 (2.30) |  |
| Rash vesicular (PT: 10037898) | 3 | 29.89 (9.62-92.88) | 29.83 (83.43) | 4.90 (0.41) | 29.77 (9.58) |  |
| Skin hyperpigmentation (PT: 10040865) | 3 | 11.96 (3.85-37.13) | 11.93 (30.04) | 3.58 (0.23) | 11.93 (3.84) |  |
| **Hepatobiliary disorders (SOC: 10019805)** | | | | | | |
| Hepatocellular injury (PT: 10019837) | 26 | 68.35 (46.34-100.83) | 67.12 (1686.82) | 6.06 (3.72) | 66.84 (45.31) |  |
| Hepatic cytolysis (PT: 10049199) | 22 | 63.31 (41.51-96.54) | 62.34 (1323.02) | 5.96 (3.48) | 62.10 (40.72) |  |
| Cholestasis (PT: 10008635) | 14 | 33.10 (19.54-56.06) | 32.78 (430.63) | 5.03 (2.64) | 32.72 (19.32) |  |
| Hepatomegaly (PT: 10019842) | 7 | 41.14 (19.56-86.53) | 40.94 (272.08) | 5.35 (1.75) | 40.84 (19.41) |  |
| Mixed liver injury (PT: 10066758) | 6 | 80.51 (36.03-179.86) | 80.17 (466.78) | 6.32 (1.61) | 79.78 (35.71) |  |
| Hepatic failure (PT: 10019663) | 6 | 11.51 (5.16-25.67) | 11.47 (57.30) | 3.52 (1.11) | 11.46 (5.14) |  |
| Hypertransaminasaemia (PT: 10068237) | 5 | 25.86 (10.74-62.26) | 25.77 (118.86) | 4.69 (1.15) | 25.73 (10.68) |  |
| Hyperbilirubinaemia (PT: 10020578) | 4 | 19.22 (7.20-51.30) | 19.16 (68.80) | 4.26 (0.75) | 19.14 (7.17) |  |
| Acute hepatic failure (PT: 10000804) | 3 | 10.44 (3.36-32.43) | 10.42 (25.54) | 3.38 (0.19) | 10.42 (3.35) |  |
| Hepatitis cholestatic (PT: 10019754) | 3 | 27.75 (8.93-86.23) | 27.69 (77.06) | 4.79 (0.40) | 27.65 (8.90) |  |
| **Blood and lymphatic system disorders (SOC: 10005329)** | | | | | | |
| Eosinophilia (PT: 10014950) | 25 | 62.14 (41.81-92.35) | 61.06 (1471.71) | 5.93 (3.63) | 60.83 (40.93) |  |
| Thrombocytopenia (PT: 10043554) | 14 | 5.87 (3.47-9.93) | 5.82 (55.95) | 2.54 (1.39) | 5.82 (3.44) |  |
| Neutropenia (PT: 10029354) | 13 | 3.58 (2.07-6.18) | 3.56 (23.94) | 1.83 (0.81) | 3.56 (2.06) |  |
| Agranulocytosis (PT: 10001507) | 11 | 27.87 (15.39-50.46) | 27.66 (282.24) | 4.79 (2.26) | 27.61 (15.25) |  |
| Lymphadenopathy (PT: 10025197) | 6 | 8.03 (3.60-17.90) | 8.00 (36.73) | 3.00 (0.91) | 7.99 (3.58) |  |
| Bicytopenia (PT: 10058956) | 4 | 68.52 (25.63-183.22) | 68.33 (264.28) | 6.09 (0.94) | 68.05 (25.45) |  |
| Lymphopenia (PT: 10025327) | 4 | 10.80 (4.04-28.82) | 10.77 (35.43) | 3.43 (0.57) | 10.76 (4.03) |  |
| Heparin-induced thrombocytopenia (PT: 10062506) | 3 | 37.80 (12.16-117.51) | 37.72 (107.00) | 5.23 (0.44) | 37.64 (12.11) |  |
| **Investigations (SOC: 10022891)** | | | | | | |
| Aspartate aminotransferase increased (PT: 10003481) | 13 | 14.38 (8.33-24.83) | 14.26 (160.19) | 3.83 (2.10) | 14.24 (8.25) |  |
| Alanine aminotransferase increased (PT: 10001551) | 12 | 10.85 (6.15-19.16) | 10.77 (106.36) | 3.43 (1.82) | 10.76 (6.10) |  |
| Gamma-glutamyltransferase increased (PT: 10017693) | 9 | 24.37 (12.64-46.95) | 24.22 (200.07) | 4.60 (1.95) | 24.18 (12.55) |  |
| Blood lactate dehydrogenase increased (PT: 10005630) | 7 | 27.31 (12.99-57.42) | 27.18 (176.23) | 4.76 (1.64) | 27.13 (12.90) |  |
| Transaminases increased (PT: 10054889) | 6 | 11.94 (5.35-26.63) | 11.89 (59.85) | 3.57 (1.12) | 11.89 (5.33) |  |
| C-reactive protein increased (PT: 10006825) | 5 | 5.29 (2.20-12.74) | 5.28 (17.35) | 2.40 (0.44) | 5.28 (2.19) |  |
| International normalised ratio increased (PT: 10022595) | 4 | 13.70 (5.13-36.56) | 13.66 (46.91) | 3.77 (0.66) | 13.65 (5.11) |  |
| Red blood cell sedimentation rate increased (PT: 10049187) | 4 | 13.84 (5.19-36.95) | 13.81 (47.48) | 3.79 (0.66) | 13.79 (5.17) |  |
| Serum ferritin increased (PT: 10040250) | 4 | 39.05 (14.62-104.33) | 38.95 (147.54) | 5.28 (0.89) | 38.86 (14.55) |  |
| Blood alkaline phosphatase increased (PT: 10059570) | 3 | 8.17 (2.63-25.37) | 8.16 (18.83) | 3.03 (0.10) | 8.15 (2.63) |  |
| **Immune system disorders (SOC: 10021428)** | | | | | | |
| Anaphylactic shock (PT: 10002199) | 7 | 13.59 (6.46-28.56) | 13.53 (81.16) | 3.76 (1.37) | 13.52 (6.43) |  |
| Haemophagocytic lymphohistiocytosis (PT: 10071583) | 5 | 19.27 (8.00-46.39) | 19.20 (86.19) | 4.26 (1.07) | 19.18 (7.97) |  |
| **Nervous system disorders (SOC: 10029205)** | | | | | | |
| Lethargy (PT: 10024264) | 6 | 5.87 (2.63-13.08) | 5.84 (24.10) | 2.55 (0.69) | 5.84 (2.62) |  |
| Hypotonia (PT: 10021118) | 6 | 36.49 (16.35-81.43) | 36.34 (205.76) | 5.18 (1.49) | 36.26 (16.25) |  |
| Intraventricular haemorrhage (PT: 10022840) | 3 | 59.12 (19.00-183.91) | 59.00 (170.41) | 5.88 (0.48) | 58.78 (18.90) |  |
| Hydrocephalus (PT: 10020508) | 3 | 29.69 (9.55-92.25) | 29.62 (82.83) | 4.89 (0.41) | 29.57 (9.52) |  |
| **Pregnancy, puerperium and perinatal conditions (SOC: 10036585)** | | | | | | |
| Premature baby (PT: 10036590) | 5 | 6.64 (2.76-15.99) | 6.62 (23.87) | 2.73 (0.59) | 6.62 (2.75) |  |
| **General disorders and administration site conditions (SOC: 10018065)** | | | | | | |
| Oedema (PT: 10030095) | 5 | 5.06 (2.10-12.18) | 5.05 (16.24) | 2.34 (0.41) | 5.05 (2.10) |  |
| Multiple organ dysfunction syndrome (PT: 10077361) | 5 | 5.91 (2.45-14.22) | 5.89 (20.31) | 2.56 (0.52) | 5.89 (2.45) |  |
| **Vascular disorders (SOC: 10047065)** | | | | | | |
| Shock haemorrhagic (PT: 10049771) | 5 | 27.39 (11.37-65.96) | 27.30 (126.47) | 4.77 (1.16) | 27.25 (11.32) |  |
| Cyanosis (PT: 10011703) | 3 | 11.56 (3.72-35.91) | 11.54 (28.87) | 3.53 (0.22) | 11.53 (3.71) |  |
| **Renal and urinary disorders (SOC: 10038359)** | | | | | | |
| Proteinuria (PT: 10037032) | 4 | 8.81 (3.30-23.52) | 8.79 (27.61) | 3.14 (0.49) | 8.79 (3.29) |  |
| **Metabolism and nutrition disorders (SOC: 10027433)** | | | | | | |
| Hyperkalaemia (PT: 10020646) | 4 | 5.70 (2.14-15.21) | 5.69 (15.46) | 2.51 (0.26) | 5.69 (2.13) |  |
| **Respiratory, thoracic and mediastinal disorders (SOC: 10038738)** | | | | | | |
| Apnoea (PT: 10002974) | 4 | 30.21 (11.31-80.69) | 30.13 (112.46) | 4.91 (0.85) | 30.08 (11.26) |  |

**Abbreviation:** PT, preferred term; ROR, reporting odds ratio; CI, confidence interval; PRR, proportional reporting ratio; χ2, chi-squared; IC, information component; IC025, lower limit of 95% confidence interval of IC; EBGM, empirical Bayesian geometric mean; EBGM05, lower limit of 95% confidence interval of EBGM.

**Supplementary Table S9**. Number and signal strength of 59 cefotaxime-associated signals at the PT level after excluding reports with commonly co-administered drug metronidazole.

| **PT** | **Number** | **ROR (95% CI)** | **PRR (χ2)** | **IC (IC025)** | **EBGM (EBGM05)** |
| --- | --- | --- | --- | --- | --- |
| **Skin and subcutaneous tissue disorders (SOC: 10040785)** | | | | | |
| Drug reaction with eosinophilia and systemic symptoms (PT: 10073508) | 39 | 69.59 (50.56-95.80) | 67.38 (2542.64) | 6.07 (4.20) | 67.15 (48.78) |
| Rash (PT: 10037844) | 35 | 4.05 (2.90-5.67) | 3.96 (78.15) | 1.99 (1.38) | 3.96 (2.83) |
| Rash maculo-papular (PT: 10037868) | 28 | 68.92 (47.35-100.32) | 67.35 (1824.29) | 6.07 (3.81) | 67.11 (46.11) |
| Erythema (PT: 10015150) | 16 | 3.54 (2.16-5.80) | 3.51 (28.75) | 1.81 (0.91) | 3.50 (2.14) |
| Toxic epidermal necrolysis (PT: 10044223) | 12 | 47.55 (26.91-84.02) | 47.09 (540.08) | 5.55 (2.57) | 46.97 (26.58) |
| Purpura (PT: 10037549) | 12 | 90.82 (51.36-160.58) | 89.93 (1050.32) | 6.48 (2.71) | 89.50 (50.62) |
| Dermatitis exfoliative generalised (PT: 10012456) | 10 | 97.63 (52.31-182.22) | 96.83 (943.66) | 6.59 (2.44) | 96.34 (51.62) |
| Acute generalised exanthematous pustulosis (PT: 10048799) | 9 | 56.10 (29.09-108.20) | 55.70 (482.05) | 5.80 (2.19) | 55.53 (28.80) |
| Drug eruption (PT: 10013687) | 9 | 27.90 (14.47-53.77) | 27.70 (231.32) | 4.79 (2.00) | 27.66 (14.35) |
| Toxic skin eruption (PT: 10057970) | 8 | 46.16 (23.01-92.59) | 45.86 (350.25) | 5.52 (1.97) | 45.75 (22.81) |
| Rash erythematous (PT: 10037855) | 6 | 7.55 (3.38-16.84) | 7.52 (33.90) | 2.91 (0.87) | 7.51 (3.37) |
| Stevens-Johnson syndrome (PT: 10042033) | 6 | 21.34 (9.56-47.61) | 21.24 (115.61) | 4.41 (1.35) | 21.22 (9.51) |
| Petechiae (PT: 10034754) | 5 | 28.23 (11.72-68.00) | 28.12 (130.61) | 4.81 (1.17) | 28.08 (11.66) |
| Rash pruritic (PT: 10037884) | 5 | 5.34 (2.22-12.84) | 5.32 (17.54) | 2.41 (0.45) | 5.32 (2.21) |
| Skin reaction (PT: 10040914) | 4 | 15.53 (5.82-41.46) | 15.48 (54.15) | 3.95 (0.70) | 15.47 (5.79) |
| Cutaneous vasculitis (PT: 10011686) | 4 | 50.72 (18.98-135.54) | 50.56 (193.80) | 5.66 (0.92) | 50.42 (18.87) |
| Umbilical erythema (PT: 10055029) | 4 | 4428.02 (1487.78-13178.96) | 4413.40 (14284.54) | 11.80 (0.89) | 3572.94 (1200.48) |
| Skin lesion (PT: 10040882) | 4 | 7.22 (2.70-19.27) | 7.20 (21.35) | 2.85 (0.39) | 7.20 (2.70) |
| Skin hyperpigmentation (PT: 10040865) | 3 | 14.02 (4.51-43.54) | 13.98 (36.15) | 3.80 (0.27) | 13.97 (4.50) |
| Rash morbilliform (PT: 10037870) | 3 | 60.07 (19.31-186.86) | 59.93 (173.28) | 5.90 (0.48) | 59.74 (19.20) |
| Rash vesicular (PT: 10037898) | 3 | 35.04 (11.27-108.90) | 34.95 (98.76) | 5.12 (0.43) | 34.89 (11.22) |
| **Hepatobiliary disorders (SOC: 10019805)** | | | | | |
| Hepatocellular injury (PT: 10019837) | 21 | 64.57 (41.91-99.48) | 63.47 (1287.20) | 5.98 (3.42) | 63.26 (41.06) |
| Hepatic cytolysis (PT: 10049199) | 16 | 53.77 (32.81-88.11) | 53.07 (815.32) | 5.73 (3.00) | 52.92 (32.29) |
| Cholestasis (PT: 10008635) | 9 | 24.85 (12.89-47.90) | 24.68 (204.25) | 4.62 (1.96) | 24.65 (12.79) |
| Hepatomegaly (PT: 10019842) | 7 | 48.24 (22.93-101.51) | 47.97 (321.17) | 5.58 (1.78) | 47.85 (22.74) |
| Mixed liver injury (PT: 10066758) | 5 | 78.54 (32.57-189.38) | 78.22 (379.60) | 6.28 (1.31) | 77.90 (32.31) |
| Hepatic failure (PT: 10019663) | 5 | 11.24 (4.67-27.06) | 11.20 (46.41) | 3.48 (0.87) | 11.19 (4.65) |
| Hypertransaminasaemia (PT: 10068237) | 4 | 24.22 (9.07-64.69) | 24.15 (88.66) | 4.59 (0.81) | 24.12 (9.03) |
| Acute hepatic failure (PT: 10000804) | 3 | 12.24 (3.94-38.02) | 12.21 (30.87) | 3.61 (0.24) | 12.20 (3.93) |
| Hyperbilirubinaemia (PT: 10020578) | 3 | 16.88 (5.43-52.43) | 16.84 (44.66) | 4.07 (0.32) | 16.82 (5.42) |
| **Investigations (SOC: 10022891)** | | | | | |
| Aspartate aminotransferase increased (PT: 10003481) | 13 | 16.87 (9.77-29.15) | 16.70 (191.88) | 4.06 (2.20) | 16.69 (9.66) |
| Alanine aminotransferase increased (PT: 10001551) | 12 | 12.74 (7.21-22.49) | 12.62 (128.40) | 3.66 (1.93) | 12.61 (7.14) |
| Blood lactate dehydrogenase increased (PT: 10005630) | 7 | 32.02 (15.22-67.36) | 31.85 (208.82) | 4.99 (1.69) | 31.79 (15.11) |
| Gamma-glutamyltransferase increased (PT: 10017693) | 6 | 19.00 (8.51-42.39) | 18.91 (101.69) | 4.24 (1.31) | 18.89 (8.47) |
| Transaminases increased (PT: 10054889) | 5 | 11.66 (4.84-28.06) | 11.61 (48.48) | 3.54 (0.89) | 11.61 (4.82) |
| C-reactive protein increased (PT: 10006825) | 5 | 6.21 (2.58-14.94) | 6.19 (21.75) | 2.63 (0.55) | 6.18 (2.57) |
| International normalised ratio increased (PT: 10022595) | 4 | 16.06 (6.01-42.87) | 16.01 (56.24) | 4.00 (0.71) | 15.99 (5.99) |
| Red blood cell sedimentation rate increased (PT: 10049187) | 4 | 16.23 (6.08-43.32) | 16.18 (56.92) | 4.01 (0.71) | 16.16 (6.05) |
| Serum ferritin increased (PT: 10040250) | 4 | 45.79 (17.14-122.34) | 45.64 (174.23) | 5.51 (0.91) | 45.53 (17.04) |
| **Blood and lymphatic system disorders (SOC: 10005329)** | | | | | |
| Eosinophilia (PT: 10014950) | 24 | 70.04 (46.73-105.00) | 68.68 (1595.18) | 6.10 (3.63) | 68.43 (45.65) |
| Thrombocytopenia (PT: 10043554) | 12 | 5.89 (3.34-10.41) | 5.84 (48.25) | 2.55 (1.28) | 5.84 (3.31) |
| Agranulocytosis (PT: 10001507) | 5 | 14.78 (6.14-35.57) | 14.72 (63.90) | 3.88 (0.98) | 14.71 (6.11) |
| Lymphadenopathy (PT: 10025197) | 5 | 7.84 (3.25-18.86) | 7.81 (29.68) | 2.96 (0.69) | 7.80 (3.24) |
| Bicytopenia (PT: 10058956) | 4 | 80.33 (30.04-214.84) | 80.07 (311.02) | 6.32 (0.95) | 79.74 (29.82) |
| Lymphopenia (PT: 10025327) | 3 | 9.48 (3.05-29.45) | 9.46 (22.70) | 3.24 (0.16) | 9.46 (3.05) |
| **Nervous system disorders (SOC: 10029205)** | | | | | |
| Lethargy (PT: 10024264) | 6 | 6.88 (3.08-15.34) | 6.85 (29.98) | 2.78 (0.80) | 6.85 (3.07) |
| Hypotonia (PT: 10021118) | 6 | 42.79 (19.17-95.52) | 42.58 (243.10) | 5.41 (1.52) | 42.49 (19.03) |
| Hydrocephalus (PT: 10020508) | 3 | 34.80 (11.20-108.16) | 34.71 (98.05) | 5.11 (0.43) | 34.65 (11.15) |
| Intraventricular haemorrhage (PT: 10022840) | 3 | 69.30 (22.27-215.62) | 69.13 (200.70) | 6.11 (0.49) | 68.88 (22.14) |
| **Immune system disorders (SOC: 10021428)** | | | | | |
| Anaphylactic shock (PT: 10002199) | 5 | 11.36 (4.72-27.35) | 11.32 (47.02) | 3.50 (0.88) | 11.31 (4.70) |
| Haemophagocytic lymphohistiocytosis (PT: 10071583) | 3 | 13.53 (4.35-42.01) | 13.49 (34.69) | 3.75 (0.26) | 13.49 (4.34) |
| **Renal and urinary disorders (SOC: 10038359)** | | | | | |
| Proteinuria (PT: 10037032) | 4 | 10.33 (3.87-27.58) | 10.30 (33.58) | 3.36 (0.55) | 10.30 (3.86) |
| Tubulointerstitial nephritis (PT: 10048302) | 3 | 6.83 (2.20-21.22) | 6.82 (14.89) | 2.77 (0.03) | 6.82 (2.19) |
| **Vascular disorders (SOC: 10047065)** | | | | | |
| Shock haemorrhagic (PT: 10049771) | 4 | 25.66 (9.61-68.53) | 25.58 (94.36) | 4.68 (0.82) | 25.55 (9.57) |
| Cyanosis (PT: 10011703) | 3 | 13.55 (4.36-42.10) | 13.52 (34.77) | 3.76 (0.26) | 13.51 (4.35) |
| **General disorders and administration site conditions (SOC: 10018065)** | | | | | |
| Multiple organ dysfunction syndrome (PT: 10077361) | 5 | 6.93 (2.88-16.68) | 6.90 (25.24) | 2.79 (0.62) | 6.90 (2.87) |
| **Pregnancy, puerperium and perinatal conditions (SOC: 10036585)** | | | | | |
| Premature baby (PT: 10036590) | 5 | 7.79 (3.23-18.75) | 7.76 (29.45) | 2.96 (0.69) | 7.76 (3.22) |
| **Metabolism and nutrition disorders (SOC: 10027433)** | | | | | |
| Hyperkalaemia (PT: 10020646) | 4 | 6.68 (2.50-17.84) | 6.66 (19.26) | 2.74 (0.35) | 6.66 (2.50) |
| **Respiratory, thoracic and mediastinal disorders (SOC: 10038738)** | | | | | |
| Apnoea (PT: 10002974) | 4 | 35.42 (13.26-94.62) | 35.31 (133.10) | 5.14 (0.87) | 35.24 (13.19) |

**Abbreviation:** PT, preferred term; ROR, reporting odds ratio; CI, confidence interval; PRR, proportional reporting ratio; χ2, chi-squared; IC, information component; IC025, lower limit of 95% confidence interval of IC; EBGM, empirical Bayesian geometric mean; EBGM05, lower limit of 95% confidence interval of EBGM.

**Supplementary Table S10**. Number and signal strength of 60 cefotaxime-associated signals at the PT level after excluding reports with commonly co-administered drug amoxicillin.

| **PT** | **Number** | **ROR (95% CI)** | **PRR (χ2)** | **IC (IC025)** | **EBGM (EBGM05)** |
| --- | --- | --- | --- | --- | --- |
| **Skin and subcutaneous tissue disorders (SOC: 10040785)** | | | | | |
| Drug reaction with eosinophilia and systemic symptoms (PT: 10073508) | 46 | 79.56 (59.23-106.86) | 76.68 (3423.03) | 6.25 (4.44) | 76.36 (56.85) |
| Rash (PT: 10037844) | 36 | 4.02 (2.88-5.60) | 3.93 (79.27) | 1.98 (1.38) | 3.93 (2.82) |
| Rash maculo-papular (PT: 10037868) | 28 | 66.39 (45.62-96.63) | 64.94 (1756.96) | 6.02 (3.80) | 64.71 (44.46) |
| Erythema (PT: 10015150) | 16 | 3.41 (2.08-5.58) | 3.38 (26.90) | 1.76 (0.86) | 3.38 (2.06) |
| Toxic epidermal necrolysis (PT: 10044223) | 11 | 41.97 (23.17-76.03) | 41.61 (435.06) | 5.38 (2.41) | 41.52 (22.92) |
| Toxic skin eruption (PT: 10057970) | 10 | 55.74 (29.89-103.95) | 55.31 (531.69) | 5.79 (2.34) | 55.14 (29.57) |
| Purpura (PT: 10037549) | 10 | 72.77 (39.01-135.74) | 72.20 (699.38) | 6.17 (2.40) | 71.91 (38.55) |
| Dermatitis exfoliative generalised (PT: 10012456) | 9 | 84.58 (43.84-163.20) | 83.99 (734.63) | 6.39 (2.26) | 83.60 (43.33) |
| Drug eruption (PT: 10013687) | 8 | 23.88 (11.91-47.88) | 23.73 (174.02) | 4.57 (1.78) | 23.70 (11.82) |
| Rash erythematous (PT: 10037855) | 8 | 9.72 (4.85-19.48) | 9.66 (62.14) | 3.27 (1.33) | 9.66 (4.82) |
| Acute generalised exanthematous pustulosis (PT: 10048799) | 8 | 48.02 (23.94-96.32) | 47.72 (364.99) | 5.57 (1.98) | 47.59 (23.73) |
| Stevens-Johnson syndrome (PT: 10042033) | 6 | 20.57 (9.22-45.90) | 20.48 (111.06) | 4.35 (1.34) | 20.46 (9.17) |
| Rash pruritic (PT: 10037884) | 5 | 5.14 (2.14-12.38) | 5.13 (16.62) | 2.36 (0.42) | 5.13 (2.13) |
| Rash morbilliform (PT: 10037870) | 4 | 77.36 (28.93-206.89) | 77.12 (299.27) | 6.26 (0.95) | 76.80 (28.72) |
| Cutaneous vasculitis (PT: 10011686) | 4 | 48.90 (18.30-130.66) | 48.75 (186.58) | 5.60 (0.91) | 48.62 (18.19) |
| Umbilical erythema (PT: 10055029) | 4 | 4268.86 (1434.38-12704.59) | 4255.27 (13772.49) | 11.75 (0.89) | 3444.93 (1157.53) |
| Skin reaction (PT: 10040914) | 4 | 14.97 (5.61-39.97) | 14.93 (51.95) | 3.90 (0.69) | 14.92 (5.59) |
| Petechiae (PT: 10034754) | 4 | 21.75 (8.15-58.08) | 21.68 (78.84) | 4.44 (0.78) | 21.66 (8.11) |
| Skin hyperpigmentation (PT: 10040865) | 3 | 13.51 (4.35-41.97) | 13.48 (34.65) | 3.75 (0.26) | 13.47 (4.34) |
| **Hepatobiliary disorders (SOC: 10019805)** | | | | | |
| Hepatocellular injury (PT: 10019837) | 22 | 65.25 (42.77-99.53) | 64.12 (1362.54) | 6.00 (3.49) | 63.90 (41.89) |
| Hepatic cytolysis (PT: 10049199) | 19 | 61.71 (39.20-97.16) | 60.79 (1113.95) | 5.92 (3.28) | 60.59 (38.49) |
| Cholestasis (PT: 10008635) | 9 | 23.96 (12.43-46.17) | 23.79 (196.31) | 4.57 (1.94) | 23.76 (12.33) |
| Hepatomegaly (PT: 10019842) | 7 | 46.51 (22.10-97.85) | 46.25 (309.17) | 5.53 (1.77) | 46.14 (21.93) |
| Hypertransaminasaemia (PT: 10068237) | 5 | 29.23 (12.13-70.39) | 29.11 (135.54) | 4.86 (1.17) | 29.07 (12.07) |
| Mixed liver injury (PT: 10066758) | 4 | 60.47 (22.62-161.64) | 60.28 (232.44) | 5.91 (0.93) | 60.09 (22.48) |
| Hyperbilirubinaemia (PT: 10020578) | 4 | 21.72 (8.13-57.99) | 21.65 (78.71) | 4.43 (0.78) | 21.63 (8.10) |
| Acute hepatic failure (PT: 10000804) | 3 | 11.80 (3.80-36.65) | 11.77 (29.56) | 3.56 (0.23) | 11.77 (3.79) |
| Hepatic failure (PT: 10019663) | 3 | 6.49 (2.09-20.15) | 6.48 (13.89) | 2.69 (0.00) | 6.47 (2.08) |
| **Investigations (SOC: 10022891)** | | | | | |
| Aspartate aminotransferase increased (PT: 10003481) | 13 | 16.26 (9.41-28.09) | 16.11 (184.13) | 4.01 (2.18) | 16.09 (9.31) |
| Alanine aminotransferase increased (PT: 10001551) | 12 | 12.28 (6.95-21.68) | 12.17 (123.01) | 3.60 (1.90) | 12.16 (6.89) |
| Gamma-glutamyltransferase increased (PT: 10017693) | 9 | 27.55 (14.29-53.10) | 27.36 (228.28) | 4.77 (1.99) | 27.32 (14.17) |
| Blood lactate dehydrogenase increased (PT: 10005630) | 7 | 30.87 (14.68-64.93) | 30.70 (200.85) | 4.94 (1.68) | 30.65 (14.57) |
| C-reactive protein increased (PT: 10006825) | 5 | 5.98 (2.49-14.41) | 5.96 (20.67) | 2.58 (0.52) | 5.96 (2.48) |
| Transaminases increased (PT: 10054889) | 4 | 8.98 (3.36-23.97) | 8.96 (28.27) | 3.16 (0.50) | 8.95 (3.35) |
| Serum ferritin increased (PT: 10040250) | 4 | 44.14 (16.52-117.93) | 44.00 (167.70) | 5.46 (0.90) | 43.90 (16.43) |
| Red blood cell sedimentation rate increased (PT: 10049187) | 4 | 15.64 (5.86-41.77) | 15.60 (54.61) | 3.96 (0.70) | 15.58 (5.84) |
| International normalised ratio increased (PT: 10022595) | 3 | 11.60 (3.73-36.02) | 11.57 (28.97) | 3.53 (0.22) | 11.57 (3.72) |
| Blood alkaline phosphatase increased (PT: 10059570) | 3 | 9.23 (2.97-28.68) | 9.21 (21.96) | 3.20 (0.15) | 9.21 (2.97) |
| **Blood and lymphatic system disorders (SOC: 10005329)** | | | | | |
| Eosinophilia (PT: 10014950) | 21 | 58.88 (38.22-90.69) | 57.91 (1171.09) | 5.85 (3.39) | 57.73 (37.48) |
| Thrombocytopenia (PT: 10043554) | 14 | 6.64 (3.92-11.24) | 6.57 (66.26) | 2.72 (1.51) | 6.57 (3.88) |
| Agranulocytosis (PT: 10001507) | 10 | 28.62 (15.36-53.36) | 28.40 (264.05) | 4.83 (2.15) | 28.36 (15.21) |
| Lymphadenopathy (PT: 10025197) | 5 | 7.55 (3.14-18.18) | 7.53 (28.30) | 2.91 (0.67) | 7.52 (3.13) |
| Bicytopenia (PT: 10058956) | 4 | 77.45 (28.96-207.11) | 77.20 (299.60) | 6.26 (0.95) | 76.88 (28.75) |
| Lymphopenia (PT: 10025327) | 4 | 12.20 (4.57-32.57) | 12.17 (40.98) | 3.60 (0.62) | 12.16 (4.55) |
| Heparin-induced thrombocytopenia (PT: 10062506) | 3 | 42.72 (13.74-132.81) | 42.62 (121.65) | 5.41 (0.45) | 42.52 (13.68) |
| **Nervous system disorders (SOC: 10029205)** | | | | | |
| Lethargy (PT: 10024264) | 6 | 6.63 (2.97-14.79) | 6.60 (28.54) | 2.72 (0.78) | 6.60 (2.96) |
| Hypotonia (PT: 10021118) | 4 | 27.43 (10.27-73.26) | 27.35 (101.40) | 4.77 (0.83) | 27.31 (10.23) |
| Intraventricular haemorrhage (PT: 10022840) | 3 | 66.81 (21.47-207.86) | 66.65 (193.30) | 6.05 (0.49) | 66.41 (21.35) |
| Hydrocephalus (PT: 10020508) | 3 | 33.55 (10.79-104.27) | 33.47 (94.33) | 5.06 (0.43) | 33.41 (10.75) |
| **General disorders and administration site conditions (SOC: 10018065)** | | | | | |
| Multiple organ dysfunction syndrome (PT: 10077361) | 5 | 6.68 (2.77-16.07) | 6.66 (24.03) | 2.73 (0.59) | 6.65 (2.76) |
| Oedema (PT: 10030095) | 5 | 5.72 (2.38-13.77) | 5.70 (19.40) | 2.51 (0.50) | 5.70 (2.37) |
| **Immune system disorders (SOC: 10021428)** | | | | | |
| Anaphylactic shock (PT: 10002199) | 5 | 10.95 (4.55-26.37) | 10.91 (45.01) | 3.45 (0.86) | 10.91 (4.53) |
| Haemophagocytic lymphohistiocytosis (PT: 10071583) | 5 | 21.78 (9.04-52.44) | 21.69 (98.60) | 4.44 (1.10) | 21.67 (9.00) |
| **Renal and urinary disorders (SOC: 10038359)** | | | | | |
| Proteinuria (PT: 10037032) | 4 | 9.96 (3.73-26.59) | 9.93 (32.12) | 3.31 (0.54) | 9.93 (3.72) |
| Tubulointerstitial nephritis (PT: 10048302) | 3 | 6.59 (2.12-20.45) | 6.57 (14.18) | 2.72 (0.01) | 6.57 (2.12) |
| **Vascular disorders (SOC: 10047065)** | | | | | |
| Shock haemorrhagic (PT: 10049771) | 4 | 24.74 (9.26-66.06) | 24.66 (90.71) | 4.62 (0.81) | 24.63 (9.22) |
| Cyanosis (PT: 10011703) | 3 | 13.07 (4.21-40.59) | 13.04 (33.33) | 3.70 (0.26) | 13.03 (4.20) |
| **Pregnancy, puerperium and perinatal conditions (SOC: 10036585)** | | | | | |
| Premature baby (PT: 10036590) | 5 | 7.51 (3.12-18.07) | 7.48 (28.08) | 2.90 (0.66) | 7.48 (3.11) |
| **Metabolism and nutrition disorders (SOC: 10027433)** | | | | | |
| Hyperkalaemia (PT: 10020646) | 4 | 6.44 (2.41-17.20) | 6.43 (18.33) | 2.68 (0.33) | 6.42 (2.41) |
| **Respiratory, thoracic and mediastinal disorders (SOC: 10038738)** | | | | | |
| Apnoea (PT: 10002974) | 4 | 34.15 (12.78-91.21) | 34.04 (128.06) | 5.09 (0.87) | 33.98 (12.72) |

**Abbreviation:** PT, preferred term; ROR, reporting odds ratio; CI, confidence interval; PRR, proportional reporting ratio; χ2, chi-squared; IC, information component; IC025, lower limit of 95% confidence interval of IC; EBGM, empirical Bayesian geometric mean; EBGM05, lower limit of 95% confidence interval of EBGM.

**Supplementary Table S11**. Number and signal strength of 56 cefotaxime-associated signals at the PT level after excluding reports with commonly co-administered drug vancomycin.

| **PT** | **Number** | **ROR (95% CI)** | **PRR (χ2)** | **IC (IC025)** | **EBGM (EBGM05)** |
| --- | --- | --- | --- | --- | --- |
| **Skin and subcutaneous tissue disorders (SOC: 10040785)** | | | | | |
| Rash (PT: 10037844) | 37 | 4.51 (3.25-6.26) | 4.40 (97.85) | 2.14 (1.54) | 4.40 (3.17) |
| Drug reaction with eosinophilia and systemic symptoms (PT: 10073508) | 36 | 67.32 (48.28-93.87) | 65.26 (2271.30) | 6.02 (4.09) | 65.04 (46.65) |
| Rash maculo-papular (PT: 10037868) | 24 | 61.81 (41.23-92.67) | 60.55 (1401.74) | 5.92 (3.58) | 60.37 (40.27) |
| Erythema (PT: 10015150) | 16 | 3.72 (2.27-6.09) | 3.68 (31.31) | 1.88 (0.96) | 3.68 (2.25) |
| Toxic epidermal necrolysis (PT: 10044223) | 12 | 49.92 (28.25-88.23) | 49.41 (567.91) | 5.62 (2.58) | 49.29 (27.89) |
| Acute generalised exanthematous pustulosis (PT: 10048799) | 11 | 72.16 (39.81-130.81) | 71.48 (761.79) | 6.15 (2.54) | 71.23 (39.29) |
| Toxic skin eruption (PT: 10057970) | 10 | 60.71 (32.55-113.25) | 60.19 (580.42) | 5.91 (2.36) | 60.01 (32.17) |
| Drug eruption (PT: 10013687) | 9 | 29.29 (15.19-56.46) | 29.06 (243.60) | 4.86 (2.01) | 29.02 (15.06) |
| Purpura (PT: 10037549) | 9 | 71.24 (36.93-137.43) | 70.69 (616.20) | 6.14 (2.23) | 70.44 (36.51) |
| Dermatitis exfoliative generalised (PT: 10012456) | 8 | 81.77 (40.74-164.14) | 81.21 (631.23) | 6.34 (2.07) | 80.88 (40.29) |
| Rash erythematous (PT: 10037855) | 7 | 9.25 (4.40-19.45) | 9.20 (51.18) | 3.20 (1.16) | 9.20 (4.37) |
| Stevens-Johnson syndrome (PT: 10042033) | 7 | 26.16 (12.44-55.02) | 26.01 (168.13) | 4.70 (1.63) | 25.97 (12.35) |
| Skin reaction (PT: 10040914) | 5 | 20.40 (8.47-49.12) | 20.31 (91.74) | 4.34 (1.09) | 20.29 (8.43) |
| Rash pruritic (PT: 10037884) | 5 | 5.60 (2.33-13.48) | 5.58 (18.81) | 2.48 (0.48) | 5.58 (2.32) |
| Skin lesion (PT: 10040882) | 4 | 7.58 (2.84-20.22) | 7.55 (22.74) | 2.92 (0.41) | 7.55 (2.83) |
| Petechiae (PT: 10034754) | 4 | 23.68 (8.87-63.24) | 23.60 (86.48) | 4.56 (0.80) | 23.57 (8.83) |
| Dermatitis bullous (PT: 10012441) | 4 | 34.60 (12.95-92.44) | 34.49 (129.85) | 5.11 (0.87) | 34.43 (12.89) |
| Rash morbilliform (PT: 10037870) | 3 | 63.05 (20.27-196.13) | 62.89 (182.13) | 5.97 (0.48) | 62.69 (20.15) |
| Cutaneous vasculitis (PT: 10011686) | 3 | 39.87 (12.82-123.93) | 39.76 (113.15) | 5.31 (0.45) | 39.69 (12.77) |
| **Hepatobiliary disorders (SOC: 10019805)** | | | | | |
| Hepatocellular injury (PT: 10019837) | 24 | 77.75 (51.86-116.58) | 76.16 (1773.71) | 6.25 (3.66) | 75.87 (50.60) |
| Hepatic cytolysis (PT: 10049199) | 19 | 67.26 (42.71-105.92) | 66.17 (1215.71) | 6.04 (3.30) | 65.95 (41.88) |
| Cholestasis (PT: 10008635) | 12 | 34.89 (19.75-61.66) | 34.54 (390.29) | 5.11 (2.46) | 34.48 (19.52) |
| Mixed liver injury (PT: 10066758) | 6 | 99.09 (44.34-221.47) | 98.58 (576.69) | 6.62 (1.62) | 98.10 (43.89) |
| Hepatomegaly (PT: 10019842) | 5 | 36.08 (14.98-86.93) | 35.93 (169.51) | 5.16 (1.21) | 35.87 (14.89) |
| Hyperbilirubinaemia (PT: 10020578) | 4 | 23.64 (8.85-63.14) | 23.57 (86.34) | 4.56 (0.80) | 23.54 (8.81) |
| Hypertransaminasaemia (PT: 10068237) | 4 | 25.43 (9.52-67.90) | 25.34 (93.41) | 4.66 (0.82) | 25.31 (9.48) |
| Hepatitis cholestatic (PT: 10019754) | 3 | 34.14 (10.98-106.12) | 34.05 (96.09) | 5.09 (0.43) | 34.00 (10.94) |
| Acute hepatic failure (PT: 10000804) | 3 | 12.85 (4.14-39.90) | 12.81 (32.66) | 3.68 (0.25) | 12.81 (4.12) |
| Hepatic failure (PT: 10019663) | 3 | 7.06 (2.27-21.94) | 7.05 (15.57) | 2.82 (0.04) | 7.05 (2.27) |
| **Investigations (SOC: 10022891)** | | | | | |
| Aspartate aminotransferase increased (PT: 10003481) | 10 | 13.59 (7.29-25.33) | 13.48 (115.55) | 3.75 (1.78) | 13.47 (7.23) |
| Alanine aminotransferase increased (PT: 10001551) | 9 | 10.00 (5.19-19.27) | 9.93 (72.30) | 3.31 (1.47) | 9.93 (5.15) |
| Gamma-glutamyltransferase increased (PT: 10017693) | 8 | 26.64 (13.29-53.43) | 26.47 (195.81) | 4.72 (1.82) | 26.43 (13.18) |
| Blood lactate dehydrogenase increased (PT: 10005630) | 7 | 33.62 (15.98-70.72) | 33.42 (219.81) | 5.06 (1.70) | 33.36 (15.86) |
| C-reactive protein increased (PT: 10006825) | 5 | 6.52 (2.71-15.69) | 6.49 (23.24) | 2.70 (0.58) | 6.49 (2.70) |
| Red blood cell sedimentation rate increased (PT: 10049187) | 4 | 17.03 (6.38-45.48) | 16.98 (60.10) | 4.08 (0.72) | 16.96 (6.35) |
| International normalised ratio increased (PT: 10022595) | 4 | 16.85 (6.31-45.00) | 16.80 (59.39) | 4.07 (0.72) | 16.78 (6.29) |
| Transaminases increased (PT: 10054889) | 4 | 9.78 (3.66-26.10) | 9.75 (31.40) | 3.28 (0.53) | 9.74 (3.65) |
| Serum ferritin increased (PT: 10040250) | 4 | 48.05 (17.98-128.41) | 47.89 (183.22) | 5.58 (0.91) | 47.78 (17.88) |
| Blood alkaline phosphatase increased (PT: 10059570) | 3 | 10.05 (3.24-31.22) | 10.03 (24.38) | 3.33 (0.18) | 10.02 (3.23) |
| **Blood and lymphatic system disorders (SOC: 10005329)** | | | | | |
| Eosinophilia (PT: 10014950) | 17 | 51.74 (32.03-83.58) | 50.99 (831.31) | 5.67 (3.07) | 50.86 (31.49) |
| Thrombocytopenia (PT: 10043554) | 14 | 7.23 (4.27-12.25) | 7.16 (74.23) | 2.84 (1.59) | 7.15 (4.22) |
| Agranulocytosis (PT: 10001507) | 9 | 28.03 (14.54-54.03) | 27.82 (232.43) | 4.80 (2.00) | 27.78 (14.41) |
| Lymphopenia (PT: 10025327) | 4 | 13.28 (4.98-35.47) | 13.24 (45.25) | 3.73 (0.65) | 13.23 (4.96) |
| Lymphadenopathy (PT: 10025197) | 4 | 6.57 (2.46-17.55) | 6.55 (18.83) | 2.71 (0.34) | 6.55 (2.45) |
| Heparin-induced thrombocytopenia (PT: 10062506) | 3 | 46.51 (14.96-144.60) | 46.39 (132.92) | 5.53 (0.46) | 46.28 (14.88) |
| Bicytopenia (PT: 10058956) | 3 | 63.11 (20.29-196.34) | 62.95 (182.33) | 5.97 (0.48) | 62.76 (20.17) |
| **Immune system disorders (SOC: 10021428)** | | | | | |
| Anaphylactic shock (PT: 10002199) | 7 | 16.73 (7.95-35.18) | 16.63 (102.80) | 4.05 (1.47) | 16.62 (7.90) |
| Anaphylactic reaction (PT: 10002198) | 5 | 5.16 (2.14-12.41) | 5.14 (16.68) | 2.36 (0.42) | 5.14 (2.13) |
| Haemophagocytic lymphohistiocytosis (PT: 10071583) | 3 | 14.20 (4.57-44.10) | 14.16 (36.67) | 3.82 (0.28) | 14.15 (4.56) |
| **Vascular disorders (SOC: 10047065)** | | | | | |
| Shock haemorrhagic (PT: 10049771) | 5 | 33.71 (13.99-81.20) | 33.57 (157.73) | 5.07 (1.20) | 33.51 (13.91) |
| Cyanosis (PT: 10011703) | 3 | 14.23 (4.58-44.19) | 14.19 (36.77) | 3.83 (0.28) | 14.18 (4.57) |
| **Renal and urinary disorders (SOC: 10038359)** | | | | | |
| Tubulointerstitial nephritis (PT: 10048302) | 3 | 7.17 (2.31-22.27) | 7.15 (15.88) | 2.84 (0.05) | 7.15 (2.30) |
| Proteinuria (PT: 10037032) | 3 | 8.12 (2.62-25.23) | 8.11 (18.69) | 3.02 (0.10) | 8.10 (2.61) |
| **General disorders and administration site conditions (SOC: 10018065)** | | | | | |
| Multiple organ dysfunction syndrome (PT: 10077361) | 5 | 7.27 (3.02-17.50) | 7.24 (26.91) | 2.86 (0.65) | 7.24 (3.01) |
| **Metabolism and nutrition disorders (SOC: 10027433)** | | | | | |
| Hyperkalaemia (PT: 10020646) | 4 | 7.02 (2.63-18.73) | 6.99 (20.55) | 2.81 (0.38) | 6.99 (2.62) |
| **Respiratory, thoracic and mediastinal disorders (SOC: 10038738)** | | | | | |
| Apnoea (PT: 10002974) | 3 | 27.85 (8.96-86.53) | 27.78 (77.33) | 4.79 (0.41) | 27.74 (8.93) |

**Abbreviation:** PT, preferred term; ROR, reporting odds ratio; CI, confidence interval; PRR, proportional reporting ratio; χ2, chi-squared; IC, information component; IC025, lower limit of 95% confidence interval of IC; EBGM, empirical Bayesian geometric mean; EBGM05, lower limit of 95% confidence interval of EBGM.


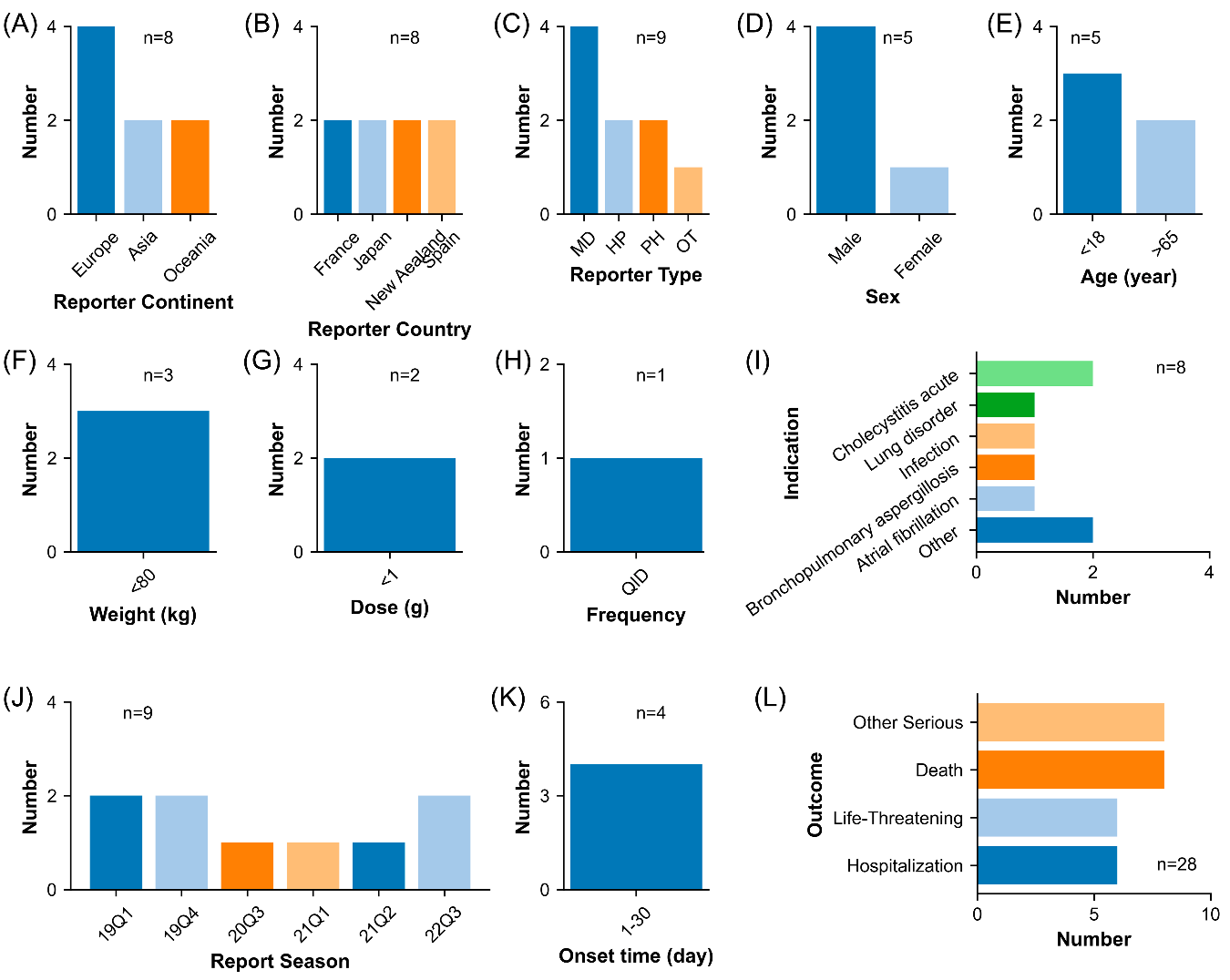


**Supplementary Figure S1.** Clinical characteristics of cefotaxime-associated “hepatic failure” and “acute hepatic failure” reports. (A) Reporter continent. (B) Reporter country. (C) Reporter type. (D) Sex. (E) Age. (F) Weight. (G) Dose. (H) Frequency. (I) Indication. (J) Report season. (K) Onset time. (L) Outcome. **Abbreviation**: MD, Physician; HP, Health-professional; PH, pharmacist; OT, other health-professional; QID, quater in die.
